# Heme Oxygenase-1 (*HMX1*) Loss of Function Increases the In-Host Fitness of the *Saccharomyces ‘boulardii’* Probiotic Yeast in a Mouse Fungemia Model

**DOI:** 10.3390/jof8050522

**Published:** 2022-05-18

**Authors:** Alexandra Imre, Renátó Kovács, Zoltán Tóth, László Majoros, Zsigmond Benkő, Walter P. Pfliegler, István Pócsi

**Affiliations:** 1 Department of Molecular Biotechnology and Microbiology, University of Debrecen, Egyetem tér 1., H4032 Debrecen, Hungary; imre.alexandra@science.unideb.hu (A.I.); benko.zsigmond@science.unideb.hu (Z.B.); pfliegler.valter@science.unideb.hu (W.P.P.); 2 Kálmán Laki Doctoral School of Biomedical and Clinical Sciences, University of Debrecen, Egyetem tér 1., H4032 Debrecen, Hungary; 3 Department of Medical Microbiology, University of Debrecen, Egyetem tér 1., H4032 Debrecen, Hungary; kovacs.renato@med.unideb.hu (R.K.); toth.zoltan@med.unideb.hu (Z.T.); major@med.unideb.hu (L.M.); 4 Faculty of Pharmacy, University of Debrecen, Egyetem tér 1., H4032 Debrecen, Hungary

**Keywords:** fungaemia, heme oxygenase, hemolysis, iron starvation, genomics, probiotic yeast, *Saccharomyces boulardii*

## Abstract

The use of yeast-containing probiotics is on the rise; however, these products occasionally cause fungal infections and possibly even fungemia among susceptible probiotic-treated patients. The incidence of such cases is probably underestimated, which is why it is important to delve deeper into the pathomechanism and the adaptive features of *S. ‘boulardii’*. Here in this study, the potential role of the gene heme oxygenase-1 (*HMX1*) in probiotic yeast bloodstream-derived infections was studied by generating marker-free *HMX1* deletion mutants with CRISPR/Cas9 technology from both commercial and clinical *S. ‘boulardii’* isolates. The six commercial and clinical yeasts used here represented closely related but different genetic backgrounds as revealed by comparative genomic analysis. We compared the wild-type isolates against deletion mutants for their tolerance of iron starvation, hemolytic activity, as well as kidney burden in immunosuppressed BALB/c mice after lateral tail vein injection. Our results reveal that the lack of *HMX1* in *S. ‘boulardii’* significantly (*p* < 0.0001) increases the kidney burden of the mice in most genetic backgrounds, while at the same time causes decreased growth in iron-deprived media in vitro. These findings indicate that even a single-gene loss-of-function mutation can, surprisingly, cause elevated fitness in the host during an opportunistic systemic infection. Our findings indicate that the safety assessment of *S. ‘boulardii’* strains should not only take strain-to-strain variation into account, but also avoid extrapolating in vitro results to in vivo virulence factor determination.

## 1. Introduction

### 1.1. Probiotic Yeast as a Cause of Fungemia

Despite the fact that *Saccharomyces* *cerevisiae* is a GRAS (generally recognized as safe) organism, it is also considered now as an emerging opportunistic pathogen [1,2,3,4]. A number of studies reported and analyzed *Saccharomyces* isolates with clinical origin. A considerable number of these were hemoculture isolates [5,6]. Not entirely surprisingly, it was revealed that most of these fungemia cases are caused by the probiotic yeast *Saccharomyces cerevisiae* var. *‘boulardii’* (*Saccharomyces ‘boulardii’*) [6], as it is a widely used probiotic supplement [7] that is known to be able to occasionally lead to bloodstream infections in susceptible patients. For example, in a recent study, the incidence of *S. ‘boulardii’* fungaemia was determined in a hospital where yeast probiotics were administered regularly for the prevention of nosocomial *Clostridioides difficile* infections. The occurrence of mycosis was 1.7 cases per 10,000 patient-days among patients treated with *S. ‘boulardii’*, which is comparable in magnitude to hospital-onset candidemia with 1.03 cases per 10,000 patient-days [8,9]. Thereof, *S. ‘boulardii’* fungaemia cases should not be overlooked. However, the pathomechanism of such infections and the virulence factors facilitating the persistence of probiotic yeasts in the bloodstream and their colonization of organs in systemic infection are still unclear and under-researched. To understand the pathomechanism of probiotic yeast systemic infections, a complex approach is necessary involving the investigation of the human immune system and intestinal barrier, and the epidemiology, virulence factors, and physiology of *S. ‘boulardii’.* Environmental factors experienced by the probiotic yeast in the host and its response to them are also crucial during pathogenicity, as shown in our recent study comparing commercial probiotic and clinical *S. ‘boulardii’* isolates [10].

### 1.2. The Role of Iron during Pathogenicity

As in every eukaryotic organism, iron plays a critical role in humans and functions as a cofactor in metalloproteins involved in the electron transport chain, oxygen transport, DNA synthesis and repair, and many others [11]. However, in cases of iron overload, toxic processes are initiated, as iron catalyzes the formation of hydroxyl radicals through the Fenton reaction. This results in serious cellular damage [12,13]. To prevent such a scenario, iron regulatory mechanisms have evolved that not only aim to prevent cytotoxicity [14], but serve as a form of nutritional immune response in case of infections to fight against pathogens as well. With the appearance of inflammatory agents or pathogens, hepcidin is released in the liver, neutrophils, and macrophages, which induces the reduction of iron levels available at the infectious loci by the removal of the cellular iron exporter ferroportin (FPN) on the cell surface [15,16,17,18]. Hepcidin-independent pathways also inhibit the uptake of iron by pathogens induced by cytokines IFN-γ, TNF-α, IL-1, and IL-6 [19,20]. Additionally, lactoferrin is present in mucosal secretions and released by neutrophils, which limits iron availability both on mucosal surfaces and at the site of infection actively [21]. Blood distributes iron to the tissues, but this iron is bound to the iron-transport protein transferrin; hence, free iron is completely absent in it under non-pathological conditions [14]. These pathways all contribute to bring forth an iron-deficient environment around pathogens. It is known that iron availability in the environment is a critical factor for the survival of pathogenic fungi [12], and *Saccharomyces* species, including the probiotic yeast, are no exception. During iron scarcity, the yeast Aft1/Aft2 transcription factors activate the members of the iron regulon, intended to enhance the acquisition of extracellular iron and mobilize intracellular iron [22].

### 1.3. Heme-Iron Utilization by the Enzyme Heme Oxygenase-1 (Hmx1)

**Figure 1:**
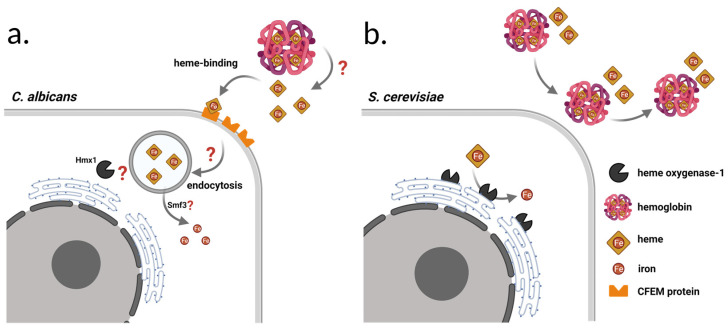
Schematic depiction of free heme and hemoglobin-heme acquisition and utilization in *C. albicans* and *S. cerevisiae*: (**a**) *C. albicans* have heme-binding, GPI anchored CFEM cell surface proteins, with which uptake of external heme is possible through endocytosis. CaHmx1 is able to break down heme; however, several steps of heme-iron utilization have not yet been fully understood (indicated by question marks); (**b**) *S. cerevisiae* does not have heme-binding cell surface molecules, and thus it is unable to utilize external free heme or hemoglobin-heme. In this yeast, Hmx1 is an ER-bound enzyme that utilizes intracellular heme as an iron source.

One important part of this iron regulon system is the endoplasmic reticulum-bound iron recycling protein called Hmx1, encoded by the gene *HMX1* (chromosome XII), which has heme oxygenase activity and catalyzes the degradation of heme into CO, biliverdin, and free iron (Figure 1b) [23,24]. It consists of 317 amino acids and has orthologs in *Candida* species, including *C. albicans*, *C. auris*, *C. dubliniensis*, *C. glabrata*, and *C. parapsilosis* [23,25,26,27]. In *C. albicans*, it has been reported that the activity of *HMX1* has a major contribution to its pathogenicity. This may be in relation to the utilization of iron directly, from host heme and heme proteins such as hemoglobin [28], but recently, CaHmx1’s role was found to be more centered around heme detoxification in *C. albicans* [29]. The localization of CaHmx1 in the cell is still unclear, and other enzymes involved in free heme and hemoglobin heme utilization have not yet been determined either (Figure 1a) [29,30,31]. The binding and uptake of free heme and hemoglobin-heme is possible through CFEM (common in several fungal extracellular membrane proteins) domain-containing proteins, namely Rbt5, Rbt51/Pga10, Pga7, and Csa2 [30,31,32]. Based on genetic and biochemical data, heme is extracted from hemoglobin extracellularly, then transferred by the CFEM protein cascade to the endocytic system. As a result, heme enters the endosome/vacuole compartment, where iron is released, and is presumably exported by the Smf3 transporter protein (Figure 1a) [30].

Though *S. cerevisiae* Hmx1 heme degradation activity has long been known, its function has not yet been linked to the pathogenicity of the yeast, presumably because of the underestimation of health risks that this fungus might pose. Similarly to other *S. cerevisiae*, *S. ‘boulardii’* also carries the gene *HMX1*, though according to our previous study it is longer than the S288c reference gene by encoding an extra serine amino acid [6].

### 1.4. Hemolytic Activity of S. cerevisiae

Putative hemolysin production of *C. albicans* highly facilitates the uptake of hemoglobin-heme by releasing hemoglobin from red blood cells by hemolysis [33,34,35]. In *S. cerevisiae*, hemolysis is considered rare, though previous studies show that alcohol stress induces the elaboration of hydrogen peroxide and lytic lipids, which might contribute to damaging the red blood cells [36,37]. Heme-binding proteins are not present in *S. cerevisiae*; therefore, it is not able to take free heme or hemoglobin-hem up as an iron source [22,38] (Figure 1b). Hemolytic activity has not been reported in the literature in the case of *S. ‘boulardii’*; however, it should be noted that studies in this regard used tryptic soy or Columbia agar-based blood medium, which are primarily suitable for the growth of bacteria. Applying such media for culturing yeasts possibly causes suboptimal growth and metabolic activity that consequently distorts hemolysis results [39,40].

### 1.5. A potential Role of HMX1 in the Pathogenicity of S. ‘boulardii’

Considering the virulence factor role of CaHmx1, the relatively frequent systemic infection of patients with serious health conditions [9,41,42,43], and the *HMX1* gene’s role in pathogenicity in other yeasts, our hypothesis was that the expression of *HMX1* might be an important factor in the pathogenicity of the probiotic yeast supporting its survival in the human host during systemic infection. Utilizing the CRISPR/Cas9 system, we created homozygous *HMX1* null mutant strains from six *S. ‘boulardii’* isolates, of which two were commercial, two originated from blood, and two were clinical isolates from other niches of the human body, all collected in Hungary. These isolates have been thoroughly characterized using complex phenotyping methods, as well as pathogenicity and immunological assays in one of our previous studies, and were shown to possess variability in colony and cell phenotypes, virulence factors (including hemolysis), and in the ways they interact with epithelial models and with immune cells [10]. This variability implies genetic differences among the tested *S. ‘boulardii’* isolates arising from (in-host) microevolution. We utilized this observation in this study as it opened the possibility to examine closely related, but not identical, genetic backgrounds while testing the effects of *HMX1* deletion.

Here, using comparative genomic analysis, we first showed that the genetic backgrounds of the studied isolates indeed show individual differences, and then compared the growth of the mutant and wild-type yeasts under iron starvation in synthetic medium and in human serum that lacks free iron under physiological conditions [14]. We then assessed their fitness in the host during infection by using an immunosuppressed BALB/c mice model and recording kidney burden. Additionally, since hemolytic activity is a known virulence factor in *C. albicans*, and is also investigated as an aspect of safety in probiotic yeasts [39,40], we measured hemolytic indices (HI) for the probiotic yeast isolates and deletion mutants used in this study. However, in contrast to some previous *S. ‘boulardii’* studies, we applied a Sabouraud-based blood agar that is widely used in hemolysis experiments in the case of pathogenic fungi, such as *C. albicans* [35,44].

## 2. Materials and Methods

### 2.1. Isolates, Mutant Strains, and Patient Data

Commercial isolates used in this study were originated from two batches of a locally available *S. ’boulardii’* CNCM I-745 probiotic supplement, to consider inter-batch variations. Clinical isolates were collected from university clinics from Debrecen and Szeged. Detailed patient and isolation data are available for these isolates (Table 1) [10]. Patient data were handled in accordance with EU, state, and local regulations with a clinical study ethics approval from the Regional and Institutional Research Ethics Council of Debrecen (DE RKEB/IKEB 5194-2019). The *ΔΔHMX1* mutant strains were made by using the CRISPR/Cas9 gene editing technology from the commercial and clinical yeast isolates used in this study. The parental isolates of each mutant strain are indicated in Table 1. Stocks of the commercial and clinical isolates and deletion mutants were saved at −70 °C in YPD broth (VWR Chemicals, Solon, OH, USA, pH 5.8) supplemented with 30% glycerol. Subculturing was minimalized in order to prevent the geno- and phenotypic changes that might be a result of clonal heterogeneity [45]. Isolates were divided into three groups according to Imre et al. [10]: a commercial (C; PY0001, PY0002), a non-mycosis (NM; 465/2018, 2251/2018), and a mycosis group (M; DE6507, DE35762). Their *ΔΔHMX1* counterparts were grouped in the same fashion into *ΔΔ*commercial (*ΔΔ*C; AI0001, AI0003), *ΔΔ*non-mycosis (*ΔΔ*NM; AI0005, AI0007), and *ΔΔ*mycosis (*ΔΔ*M; AI0009, AI0011) groups (Table 1).

### 2.2. Genome Sequencing and Assembly

Genomic DNA was isolated from the lineages after 24 h growth of the cultures following inoculation in the form of a streak on YPD agar from stocks stored at −70 °C. For Illumina sequencing, DNA isolation followed [46]. Library preparation was performed using tagmentation with the Nextera DNA Flex Library Prep kit (Illumina, San Diego, CA, USA) according to the manufacturer’s protocol, and sequencing was performed using 150 bp paired-end reads on an Illumina NextSeq 500 system, with approximately 50× coverage of the nuclear genome for PY0002, 465/2018, 2251/2018, DE6507, and DE35762, and with approximately 400× coverage for PY0001. Raw reads were deposited to NCBI SRA under BioProject PRJNA813746 (PY0001 sequencing and assembly) and PRJNA813763 (raw Illumina reads and data of isolates). Illumina FASTQ sequencing files were trimmed and filtered using fastp [47].

For Oxford Nanopore sequencing, the genomic DNA of the PY0001 isolate was isolated with the Quick-DNA™ High Molecular Weight (HMW) MagBead Kit (Zymo Research, Irvine, CA, USA) according to the company’s instructions, except that cell lysis was not performed with lysozyme, but with R-Zymolyase (RNase-A containing zymolyase; Zymo Research, Irvine, CA, USA). Library construction was performed with a Ligation Sequencing Kit (SQK LSK109; Oxford Nanopore, Oxford, UK) and sequenced on a MinION R9.4.1 Flow Cell with approximately 550× coverage calculated for the S288c genome. Basecalling and adaptor trimming (high accuracy model) of the resulting FAST5 sequencing files were performed with the MinKNOW software provided by the company. Using the obtained FASTQ files, the genome was assembled using the LRSDAY pipeline [48]. Reads from long read sequencing were first filtered and downsampled to 60× coverage, and assembly (with LRSDAY’s Canu script) was polished with the long reads (with LRSDAY’s scripts, 2 × Racon, 2 × Medaka) and with 400× coverage reads from Illumina sequencing (2× with LRSDAY’s Pilon script). The LRSDAY pipeline was used not only to assemble the genome, but also to annotate the individual genome features (genes, centromeres, transposons, telomeres) to explore the gene pool and Ty transposon types of the PY0001 isolate.

### 2.3. Comparative Genomic Analysis

The Illumina reads were mapped to the PY0001 reference genome. Mapping was performed using BWA 0.7.17. [49]. Sorted BAM files were obtained using SAMtools 1.7 [50] and Picard-tools 2.23.8 was used to mark duplicated reads. We used BEDTools 2.30.0 [51] to calculate the median coverage of chromosomes in 10,000 base windows sliding every 5000 bases, and to calculate the median coverage of the whole chromosome. Plots generated from this data were corrected for ploidy to identify potential segmental duplications or large deletions and aneuploidies. Next, median coverages of all genes in the annotation of the PY0001 assembly were calculated with BEDTools. Gene copy number variations were inferred from this data as follows (adapted from [45]). Medians of the median coverages of the upstream and downstream 20 genes along with the genes in question (altogether 41 genes) were calculated for each gene (for the first and last 20 genes of each PY0001 chromosome, medians of the median coverages of the first and last 41 genes of the chromosome, respectively, were used). Then, the ratio of the gene in question to the 41 local genes was calculated and used as an estimation of gene copy number corrected for haploid genome. Calculated gene CNVs above 1.5 were considered as 2 copies, between 0.5 and 1.5 as 1 copy. Finally, we performed a mapping to the complete pangenome ORF collection generated by Peter et al. to determine the mitochondrial, Ty transposon, rDNA, and plasmid copy numbers for the haploid genome: BEDTools was used to calculate the median coverage for each relevant gene (only nonvariable genes of the mitochondrium were considered). The median of these coverages was calculated for the mitochondrial genes and for the plasmid genes. The coverages of these genomic elements were divided by the median of chromosomal median coverages, calculated as described above. This fraction was used as the calculated copy number for the haploid genome.

Using BAM files, local realignment around indels and joint variant calling and filtering for the six samples were performed with GATK 4.1.9.0. [52,53] with regions annotated in the PY0001 reference as centromeric regions, telomeric regions, or LTRs excluded. First, genomic VCF files were obtained with the Haplotype Caller, and joint genotyping was applied. Using this initial VCF, we applied base quality score recalibration using GATK and called the BAM files again in gVCF mode. After joint calling, in the resulting VCF files, only SNPs or only INDELS were selected. SNPs were filtered according to the parameters used by Fay et al. (2019): QD < 5.0; QUAL < 30.0; SOR > 3.0; FS > 60.0; MQ < 40.0; MQRankSum < −12.5; and ReadPosRankSum < −8.0. INDELS were filtered according to the parameters QD < 5.0; QUAL < 30.0; FS > 60.0; and ReadPosRankSum < −20.0. INDELS were then left-aligned. For the final VCF files, INDELS and SNPs were merged, filtered, and non-variant sites were removed. Combined gVCF files were uploaded to FigShare (https://doi.org/10.6084/m9.figshare.19322963 (accessed on 5 March 2022)).

Variants in the individual strains were selected and exported to a.csv file using the query option of SAMtools/BCFtools 1.10.2. Allele frequency plots were obtained from these. Allele frequencies were used to estimate ploidy following [54], with the assumptions that diploids have allele ratios of approx. 1:0 or 1:1, triploids of 1:0, 1:2, and 2:1, tetraploids of 1:0, 1:3, 1:1, or 3:1, etc. The results obtained from allele ratios were compared to coverage plots to determine whether any of the isolates show deviations from the diploid euploid genome. Allele data were also used to identify allele ratio changes in the isolates compared to PY0001. First, positions called as homo- and heterozygous using the PY0001 short read mapping were exported from the VCF file and all other isolates’ homo- and heterozygous positions were compared with these data. Longer tracts of allele ratio changes, containing at least 10 consecutive positions in a given isolate that were homozygous, but heterozygous in the PY0001 genome, or vice versa, were listed. These were considered as losses and gains of heterozygous regions in the comparison to PY0001. In tracts exceeding 20 positions, one intermittent position was allowed to deviate from the gain/loss classification.

We then used the annotation file of the PY0001 genome to find mutations in genes that affect protein function using SNPEff [55]. VCF files were annotated with this software and high- and medium-effect variant positions were exported to a.csv file (together with heterozygosity data) and compared across isolates. Annotated VCF files were uploaded to FigShare (https://doi.org/10.6084/m9.figshare.19322963 (accessed on 29 March 2022)). Additionally, genetic and physical interactions involving *HMX1* and its transcriptional regulators were extracted from the *Saccharomyces* Genome Database (accessed on 29 March 2022) [56,57] to allow evaluation of the data from the aspect of the heme oxygenase. Altogether, 4 genes with physical interactions and 88 with genetic interactions have been listed, along with 12 transcriptional regulators. Fischer’s exact test was applied to test whether mutations affected *HMX1*-interacting genes to a degree significantly different from non-interacting genes. GO term analysis to assess the potential enrichment of genes affected by medium- or high-effect mutations was performed on the *Saccharomyces* Genome Database (accessed on 5 March 2022) [56,57].

### 2.4. Phylogenomics

To validate earlier results on the close relatedness of the isolates with multiplex PCR genotyping, we performed phylogenomic analyses. As a control *S. ‘boulardii’* strain, we used the strain CIM, and as an outgroup, the strain ADD (belonging to the Wine/Y-prime amplification group) from Peter et al. Joint genotyping and filtering were performed with the six isolates and these two additional genomes as described above to obtain filtered VCF files with eight genomes. The SNP VCF files were used to produce genotype matrices using vcf2phylip [58] that contained heterozygous IUPAC codes. This matrix containing heterozygosities was used in SplitsTree SplitsTree4 V4.17.0 [59] (Huson and Bryant, 2006) to create Neighbor Nets with an uncorrected P distance and averaging heterozygous positions. The matrix was also used for maximum likelihood analysis: ModelFinder [60] was applied with default setups and IQTree2 [61] was used with 1000 ultrafast bootstrap approximations. As a SNP matrix was used, ascertainment bias correction (+ASC) was applied. iTOL was used to visualize the output [62].

### 2.5. Plasmids and Constructs for HMX1 Deletion

To obtain homozygous *HMX1* null mutant strains from the *S. ‘boulardii’* isolates, the elements of the CRISPR/Cas9 system were designed and assembled using the Benchling web tool [63] and the MoClo Yeast Toolkit from Addgene (YTK, Addgene kit, Addgene, Watertown, MA, USA; catalog number: 1000000061) [64,65]. Preparation of the repair DNA was accomplished by PCR reaction using Dream Taq Green polymerase (Thermo Fisher Scientific, Waltham, MA, USA). The repair DNA was designed to knock out the *HMX1* gene and it included the 20 bp insertion cassette. Guidelines for design and preparation for the repair DNA and oligonucleotides with the gRNA sequence were followed according to Akhmetov et al. [65]. Oligonucleotide sequences of repair DNA and gRNA are presented in Table 2. The Cas9/gRNA expressing plasmid was assembled by a Golden Gate reaction combining three plasmids: an sgRNA cassette plasmid, a Cas9 cassette plasmid, and a multi-gene vector containing the nourseothricin yeast selection marker [64,65]. The sgRNA cassette plasmid was assembled using the pYTK003, pYTK072, and PYTK095 plasmids, and the pYTK050-sgRNA part plasmid, which was constructed with a Golden Gate reaction from the plasmid pYTK050 and the annealed gRNA oligo. The Cas9 cassette plasmid was constructed from the pYTK002, pYTK011, pYTK036, pYTK054, pYTK067, and pYTK095 plasmids. The multi-gene vector carrying the nourseothricin selection marker was assembled from the pYTK008, pYTK047, pYTK073, pYTK078, pYTK081, and pYTK084 plasmids. YPD plates supplemented with nourseothricin (0.1 mg/mL) were used for mutant selection. Mutants were plated on YPD agar plates (after verification of the gene deletion by PCR (see below)) to lose this plasmid, making the mutants marker-free. DH5α chemically ultra-competent *Escherichia coli* cells were used for all cloning experiments, and after transformation they were selected on lysogeny broth (LB, Miller) agar plates containing the appropriate antibiotics (ampicillin, chloramphenicol, or kanamycin). Ultra-competent *E. coli* cells were prepared with the Inoue method [66] and transformations were carried out with the heat shock method. To verify the success of gene deletion, we performed PCR with GoTaq G2 Hot Start polymerase (Promega) for each strain either to amplify the whole *HMX1* open reading frame (ORF) or to determine the correct insertion of the repair DNA cassette. The primers used for verification are listed in Table 2. For gel electrophoresis, 1% low electroendosmosis (LE) agarose gel (Promega) was used, with Tris-acetate-EDTA (TAE) buffer.

### 2.6. Yeast Transformations

Yeast transformations were performed according to Lee et al. [64], but with the following modifications: (1) prior to transformation, yeast cells were grown in 20 mL yeast peptone dextrose medium (YPD; 1% yeast extract, 2% mycological peptone, 2% glucose, 2% agar, pH 5.6); (2) for the transformation mixture (end volume is 50 µL), cells were mixed with 33.33 µL of 50% PEG-3350 (Sigma-Aldrich, St. Louis, MO, USA), 1.39 µL of boiled (95 °C, 5 min) salmon sperm DNA (Sigma-Aldrich), 5 µL of 1 M lithium acetate (pH 7.5), 4.28 µL of sterile distilled water, 200 ng of Cas9/gRNA expressing plasmid, and 1000 ng of repair DNA; (3) the transformation mixture was incubated at 30 °C for 30 min prior to the 42 °C 30 min heat shock; and (4) prior to nourseothricin resistance selection, the cells were pelleted and resuspended in YPD, and incubated at 30 °C and 220 rpm overnight. YPD agar supplemented with 0.1 mg/mL nourseothricin was used for selection of the transformants, then multiple colonies were inoculated onto YPD agar medium to promote the loss of the plasmid providing antimycotic selection.

### 2.7. Iron Starvation, Hemolytic Activity

Strains were precultured on YPD for 24 h. To investigate the effect of iron starvation, the so-called spot plate assay was used. The cell number was set to 0.5 McFarland, then isolates were inoculated in triplicates, in 4 × 10 μL droplets, each containing cells in 10× dilution series (approx. 50,000, 5000, 500, and 50 cells). To induce iron deficiency, synthetic defined (SD) agar medium (6.7 g/L yeast nitrogen base without amino acids, 2% glucose, 2% agar) was supplemented with 40, 80, and 120 µM iron chelator bathophenanthroline (BPS), and the Petri dishes were incubated at 37 °C. The SD medium without BPS is a standard medium for yeast studies, with approx. 68.86 µg/L free iron. Growth was documented photographically after 3 days. Growth in human serum, a medium lacking free iron [14], was also assessed. We obtained growth curves to compare the growth of the wild-type and *HMX1* deletion mutants. We used a medium of 50% RPMI-1640 (Sigma-Aldrich; R6504) and 50% human serum (Sigma-Aldrich; H4522) in a final volume of 5 mL. The starting inocula were 1 × 10^5^–2 × 10^5^ CFU/mL. Aliquots of 100 µL were removed at 0, 4, 8, 12, and 24 h, serially diluted 10-fold, plated onto Sabouraud dextrose agar (SDA) plates, and incubated at 35 °C for 48 h. All experiments were performed in triplicate.

For hemolysis measurements, cells were set to 1.0 McFarland in distilled water and isolates were inoculated in triplicates in 5 μL (approx. 5 × 10^4^ cells) of cell suspension in three replicates on blood Sabouraud dextrose agar (SDA) (4% glucose, 1% peptone, 7.5% sheep blood, 1.5% agar). We followed α- and β-hemolytic activity at 37 °C for 3 days in Petri dishes with good ventilation to prevent microbial alcohol-mediated hemolysis [36]. Hemolytic zones were measured after 1 and 2 (α-hemolysis) and 2 and 3 (β-hemolysis) days of incubation. We calculated HIs by dividing the zone size by the colony diameter (HI = zone size/colony diameter; HI ≥ 1).

### 2.8. Yeast Growth in Immunosuppressed BALB/c Mice

BALB/c immunocompromised female mice (21–23 g; Charles River) were used. The animals were maintained in accordance with the Guidelines for the Care and Use of Laboratory Animals. The experiments were approved by the Animal Care Committee of the University of Debrecen, Debrecen, Hungary (permission no. 12/2014 DEMÁB). Immunosuppression was achieved by intraperitoneal administration of 150 mg/kg cyclophosphamide 4 days prior to infection, 100 mg/kg cyclophosphamide 1 day prior to infection, 100 mg/kg cyclophosphamide 2 days post-infection, and 100 mg/kg cyclophosphamide 5 days post-infection. Animals were challenged intravenously through the lateral tail vein; the infectious doses were 1–1.5 × 10^7^ cells in 0.2 mL physiological saline. Inoculum density was confirmed by plating serial dilutions on Sabouraud dextrose agar. At 6 days post-infection, surviving mice were euthanized, and their kidneys were removed and homogenized aseptically.

### 2.9. Statistics and Data Visualization

To compare the results of the isolates and mutant strains, VassartStats, Astatsa, and Statistics Kingdom were used [67,68,69]. When two datasets were compared, a two-sample *t*-test (equal variances) or Welch’s test (unequal variances) were used if the datasets followed a normal distribution. In the case of a non-normal distribution, the Mann–Whitney test was used. For statistical evaluation of spot growth, the mean gray values of the spots were measured with ImageJ according to Petropavlovskiy et al. [70]. Mean gray value is the average gray value (a number specifying the intensity of gray in a particular pixel, on a scale from 0% gray (white) to 100% gray (black)) within a selected region of a photo or picture. Thus, the mean grey value is the sum of the gray values of all the pixels in the selection divided by the number of pixels. We observed that the measured mean gray values differed significantly in the case of the control SD plate when a spot was fully grown, even though there was no visible difference in growth between strains. Hence, for data normalization, the average of all measured gray values of the SD control plates was used, instead of normalization by strain. All the results were illustrated using Graphpad 9.2.0 and plotted as bar charts or violin plots. Images of iron starvation spot plate assays were cut from the original photos without manipulation. Original photos can be found in Supplementary Data uploaded to FigShare (https://doi.org/10.6084/m9.figshare.17000254.v1 (accessed on 5 March 2022)). Significant differences are marked as follows: *: *p* < 0.05; **: *p* < 0.01; ***: *p* < 0.001; and ****: *p* < 0.0001.

## 3. Results

### 3.1. Comparison of the Genetic Backgrounds of S. ‘boulardii’ Yeasts Used in This Study

To obtain information on the variations of the genetic background of the isolates used in this study, we assembled the genome of the commercial PY0001 yeast using Oxford Nanopore and Illumina sequencing. The assembly was highly syntenic with the *S. cerevisiae* reference genome and consisted of 16 chromosomes totaling 11.868 Mb (Supplementary File S1). As the mitochondrial genome was not properly assembled, we obtained the complete, circular mitochondrial assembly of the *S. ‘boulardii’* strain CIM of Peter et al. [5] and included it in our reference. Annotation uncovered 5717 nuclear putative protein coding genes, and as described for the *S. ‘boulardii’* yeasts, Ty1, Ty3, and Ty4 transposons were characteristically lacking (Supplementary File S1). This reference was used to compare all six isolates used in this study to obtain *ΔΔHMX1* mutants.

**Figure 2:**
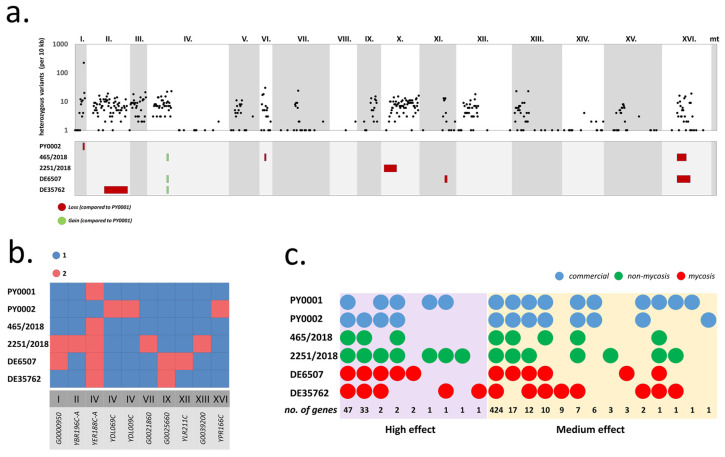
Genomic comparison of the yeast isolates used for the deletion of *HMX1*. (**a**) Heterozygous variant density (per 10 kb) across chromosomes for the isolate PY0001 (top) and changes in allele ratios, i.e., gains and losses of heterozygous regions in the other five isolates (bottom). (**b**) Gene copy number variations in the six sequenced isolates. (**c**) The distribution of protein-coding genes affected by high- and medium-effect mutations. The total number of affected genes for combinations of isolates are shown at the bottom, each genome is represented by a circle colored according to isolate type (commercial, non-mycosis, mycosis).

Phylogenomic analysis revealed that all studied yeast genomes were very closely related and differed in merely ~3000 variable sites across all six genomes, most of which were heterozygous SNPs (Supplementary File S1). Differences were the smallest between the two commercial isolates. All studied genomes were determined to be diploid euploids without segmental duplications or large deletions based on coverage and allele depth ratio data (Supplementary File S1). The most notable difference among the genomes was in regions of homo- and heterozygosity. The sequenced and assembled PY0001 isolate’s heterozygous positions mostly clustered on chromosomes I–VI (only the left arm of chr. IV), X, XII, and XIII (Figure 2a). Chromosomes overall had a low proportion of heterozygous positions (Supplementary File S1) and long runs of homozygosities were abundant in the genome; chromosomes VIII and XIV were almost completely homozygous. The genomes of the other five isolates contained one to two longer additional homozygous regions and three of them were heterozygous in a small segment of chr. IV where PY0001 was homozygous. Altogether, patterns of heterozygosities differed for all six yeast isolates (Figure 2a). Copy number variations among the yeasts were only observed for ten genes on eight different chromosomes and affected one to five genes in each strain (Figure 2b). Of these, seven were dubious or uncharacterized ORFs. Other than these, *YDL069c* (*CBS*), *YLR211C* (*ATG38*), and *YPR166C* (*MRP2*) were affected. *HMX1* was found to be a single-copy gene (per haploid genome) in all yeasts. Mitochondrial copy numbers (17 to 25) and plasmid copy numbers (31 to 58) per haploid genome were also variable, as well as the calculated number of rDNA repeats and Ty2 transposons (Supplementary File S1).

Finally, we observed differences in SNP/INDEL effects on various genes. High-effect mutations (gained/lost STOP codon, frameshift in at least one gene copy) affected altogether 90 genes when all isolates were taken into account (Figure 2c). Genes affected in PY0001 were those that possessed heterozygous mutations (note that as the reference genome used for mapping was PY0001′s own, only heterozygous variants are to be expected for this yeast genome). Of these 90 genes, 43 were not affected in all six isolates (Figure 2c, Supplementary File S1) and thus represented differences in the genetic background. Altogether, 30 of these could be assigned a well-established function based on homologies to the S288c reference genes sequences. Of the affected genes, *GLT1* and *SLX5* are known to interact with *HMX1*. The former gene had the mutation in all isolates in a heterozygous form, while *SLX5* was not affected in PY0001 and was homozygous in the other five genomes (Table 3).

The analysis of mutational effects on gene functions also recovered 497 genes (30 of which were dubious ORFs without identified S288c homologues) with moderate mutations, e.g., single amino acid substitutions. Similarly to high-effect mutations, most of the identified variants affected all isolates (most often in a heterozygous form, and in the case of PY0001, exclusively so). In total, 73 genes were affected in only the selected isolates. Genes with medium-effect mutations included *CHK1*, *ESS1*, *GLT1*, *IME1*, *KCH1*, *LGE1*, *MXR1*, *PPR1*, *SPB4*, and *CST6*, all of which have interactions with *HMX1*. In general, the medium-effect mutations in these affected all isolates in a heterozygous form, but DE35762 was homozygous for the *CHK1* mutation, and DE6507 was homozygous for the *LGE1* mutation (Table 3).

Of the altogether 11 genes in interaction with *HMX1* affected by large- or medium-effect mutations, GO Term analysis revealed enrichment in nucleus-residing proteins (*p* < 0.05) (but found no enrichment in process or in function). *HMX1*-interacting genes were not more or less likely to be affected by mutations than genes not in interactions with it (Fischer’s exact test, *p* > 0.05). Finally, *HMX1* was not affected by any medium- or high-effect mutation, and neither was its immediate upstream region affected by modifier mutations in the six isolates.

**Table 3:** List of mutations affecting genes with known interactions with *HMX1* in the six sequenced isolates. Mutations in each interacting gene are listed for all six studied genomes, heterozygous ones are marked with “het. z.”, homozygous ones with “homoz.”.

Gene Identifier	Gene Name	Description	Interaction Type	Mutation Effect	Position (on Chromosome)	Mutation Type	PY0001	PY0002	465/2018	2251/2018	DE6507	DE35762
*YBR274W*	*CHK1*	Serine/threonine kinase and DNA damage checkpoint effector	Genetic	Moderate	729,433	Missense	het. z.	het. z.	het. z.	het. z.	het. z.	homoz.
*YJR017C*	*ESS1*	Peptidylprolyl-cis/trans-isomerase (PPIase)	Genetic	Moderate	471,176	Missense	het. z.	het. z.	het. z.	het. z.	het. z.	het. z.
				Moderate	471,227	Missense	het. z.	het. z.	het. z.	het. z.	het. z.	het. z.
*YDL171C*	*GLT1*	NAD^+^-dependent glutamate synthase (GOGAT)	Genetic	High	156,574	Frameshift	het. z.	het. z.	het. z.	het. z.	het. z.	het. z.
				High	156,577	Frameshift	het. z.	het. z.	het. z.	het. z.	het. z.	het. z.
				Moderate	160,753	Missense	het. z.	het. z.	het. z.	het. z.	het. z.	het. z.
*YJR094C*	*IME1*	Master regulator of meiosis that is active only during meiotic events	Genetic	Moderate	597,121	Missense	het. z.	het. z.	het. z.	het. z.	het. z.	het. z.
				Moderate	597,137	Missense	het. z.	het. z.	het. z.	het. z.	het. z.	het. z.
				Moderate	597,835	Missense	het. z.	het. z.	het. z.	het. z.	het. z.	het. z.
*YJR054W*	*KCH1*	Potassium transporter that mediates K+ influx	Genetic	Moderate	529,412	Missense	het. z.	het. z.	het. z.	het. z.	het. z.	het. z.
				Moderate	530,009	Missense	het. z.	het. z.	het. z.	het. z.	het. z.	het. z.
				Moderate	530,230	Missense	het. z.	het. z.	het. z.	het. z.	het. z.	het. z.
*YPL055C*	*LGE1*	Protein involved in histone H2B ubiquitination	Genetic	Moderate	441,438	Missense	het. z.	het. z.	het. z.	het. z.	homoz.	het. z.
				Moderate	441,813	Missense	het. z.	het. z.	het. z.	het. z.	homoz.	het. z.
*YER042W*	*MXR1*	Methionine-S-sulfoxide reductase	Genetic	Moderate	231,971	Missense	het. z.	het. z.	het. z.	het. z.	het. z.	het. z.
*YLR014C*	*PPR1*	Zinc finger transcription factor	Genetic	Moderate	158,716	Missense	het. z.	het. z.	het. z.	het. z.	het. z.	het. z.
*YDL013W*	*SLX5*	Subunit of the Slx5-Slx8 SUMO-targeted Ub ligase (STUbL) complex	Genetic	High	436,686	Frameshift	Not present	homoz.	homoz.	homoz.	homoz.	homoz.
*YFL002C*	*SPB4*	Putative ATP-dependent RNA helicase	Genetic	Moderate	121,701	Missense	het. z.	het. z.	het. z.	het. z.	het. z.	het. z.
				Moderate	122,057	Missense	het. z.	het. z.	het. z.	het. z.	het. z.	het. z.
*YIL036W*	*CST6*	Basic leucine zipper (bZIP) transcription factor from ATF/CREB family involved in stress-responsive regulatory network	Transcription regulator	Moderate	264,026	Missense	het. z.	het. z.	het. z.	het. z.	het. z.	het. z.
				Moderate	264,770	Missense	het. z.	het. z.	het. z.	het. z.	het. z.	het. z.
				Moderate	264,849	Missense	het. z.	het. z.	het. z.	het. z.	het. z.	het. z.

### 3.2. Successful CRISPR/Cas9-Based Gene Deletion of HMX1

The deletion of the gene *HMX1* was verified with PCR by either attempting to amplify the whole *HMX1* ORF or by determining the correct insertion of the repair DNA cassette. Gel electrophoresis revealed that only the wild-type isolates carried the *HMX1* gene, which was indicated by a band 1553 bp in size. In case of the deletion mutant strains, a much shorter band of 682 bp length was visible (Supplementary Figure S1). This is because the verification primers locate upstream and downstream of the *HMX1* ORF, and hence the band size indicates the successfulness of the gene deletion. Insertion was also verified by using primers that amplified the upstream region of the insertion cassette (the forward primer annealed to –417 bp to the insertion cassette; the reverse primer annealed to the insertion cassette itself). When wild-type isolates were tested, no band was visible on the gel in contrast with the deletion strains, since a 437 bp sized band was observable (Supplementary Figure S1). Thus, gel electrophoresis revealed that the deletion was successful for all the six isolates used in this study.

### 3.3. Deletion of HMX1 Resulted in Decreased Growth under Iron Deprivation

**Figure 3:**
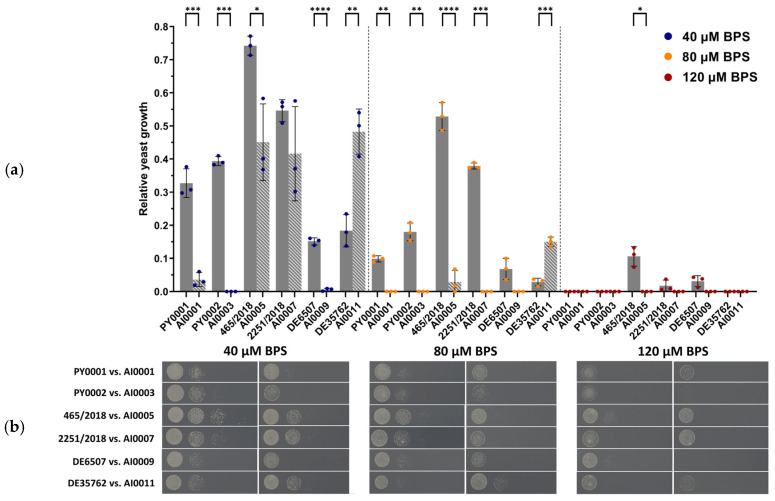
Relative yeast growth under iron starvation: (**a**) bars show the mean results for the wild type *S. ‘boulardii’* isolate triplicates (full grey bars) and for the *ΔΔHMX1* mutant strain triplicates (striped bars) (for the second spot with 5000 plated cells). Individual data points are shown in blue (40 µM BPS), orange (80 µM BPS), and red (120 µM BPS), and whiskers show the standard deviation of the data. Comparisons with significant differences are marked (*: *p* < 0.05; **: *p* < 0.01; ***: *p* < 0.001; ****: *p* < 0.0001). (**b**) Photos of iron deprivation spot plate assay. Columns represent the wild type isolate vs. *ΔΔHMX1* mutant strain pairs by BPS concentration. Raw photographs, including control plates without BPS, are uploaded to FigShare.

Previous studies have shown that *HMX1* deletion disturbs iron metabolism in *S. cerevisiae* [23,24], and under iron depletion the cells are unable to utilize internal heme-containing molecules as an iron source [24]. Thus, we controlled the mutant colonies not only with molecular methods, but we followed their growth phenotype under iron depletion as well. When the medium was supplemented with 40 µM BPS, most of the mutant strains, particularly AI0001, AI0003, AI0005, and AI0009, showed significantly (*p* < 0.001, *p* < 0.001, *p* < 0.05, and *p* < 0.001, respectively) decreased growth compared to their corresponding wild-type isolate. One of the mutant strains, namely AI0007, also appeared to be less viable compared to its parental isolate 2251/2018; however, this difference was not significant (*p* > 0.05). Interestingly, the growth of the deletion mutant AI0011 was significantly (*p* < 0.01) higher than its wild-type counterpart DE35762. When 80 µM BPS was applied, the growth of the strains AI0001, AI0003, AI0005, and AI0007 was significantly (*p* < 0.01, *p* < 0.01, *p* < 0.001, *p* < 0.001, respectively) lower considering their wild-type parental strains. The growth difference was not significant (*p* > 0.05) comparing DE6507 and AI0009, but the latter was less viable. Similarly to the previous conditions, AI0011 showed significantly (*p* < 0.001) higher growth when compared to its matching wild-type strain. Applying 120 µM BPS caused most of the strains to not grow or their growth was negligible. The isolate 465/2018 was an exception, since it showed 11% growth (normalized to the SD control). Its corresponding deletion mutant was not viable; thus, they showed a significant (*p* < 0.05) difference in growth when compared (Figure 3, Supplementary Table S1). These results suggest that the deletion of the gene *HMX1* caused a lower growth of the strains under iron starvation. Interestingly it seems this effect can be genetic background-dependent since the deletion caused increased growth during iron depletion in the case of the isolate DE35762.

**Figure 4:**
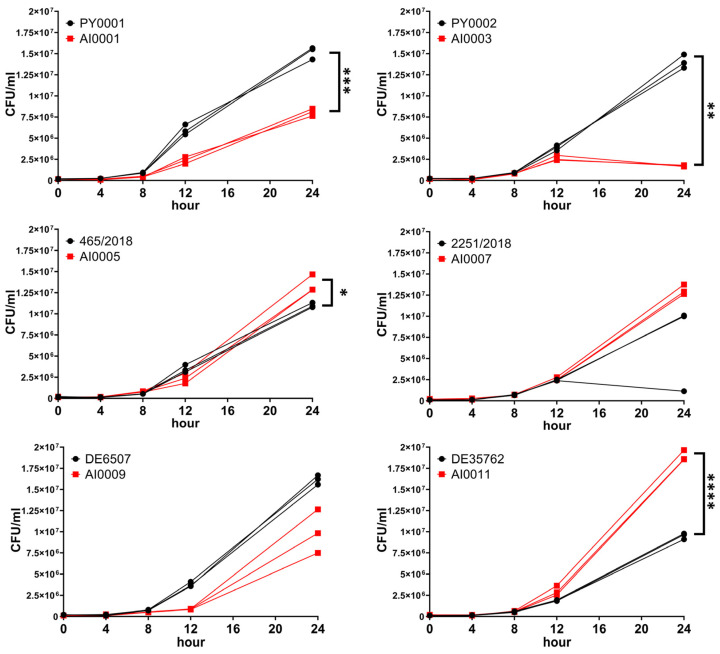
Comparison of growth curves of the isolates and *HMX1* deletion strains in medium of 50% RPMI-1640 and 50% human serum. Individual data points for wild-type strains are indicated with black, while data for the knockout mutants are indicated with red. Data points of each replicate are connected by a line. Comparisons with significant differences are marked (*: *p* < 0.05; **: *p* < 0.01; ***: *p* < 0.001; ****: *p* < 0.0001).

Besides BPS, the growth of wild-type and *HMX1* deletion mutants was also tested and compared in a liquid medium containing 50% RPMI-1640 and 50% human serum, considered as a medium free of accessible iron for the yeast. Significant difference in growth was observed in the case of PY0001, PY0002, 465/2018, and DE35762 when they were compared to their knockout counterparts (*p* < 0.001, *p* < 0.01, *p* < 0.05, and *p* < 0.0001, respectively; Figure 4, Supplementary Table S2). Interestingly, in two of these isolates (465/2018 and DE35762), the mutant strains showed a higher growth by 24 h; in the other cases, the deletion strain showed impaired growth. The isolate 2251/2018 also showed weaker growth, but it did not differ statistically from the growth of the *HMX1* mutant strain AI0007. AI0009 showed lower growth than DE6507 in all replicates; however, the difference was not significant.

### 3.4. Hemolytic Activity Was Not Affected by HMX1 Deletion

**Figure 5:**
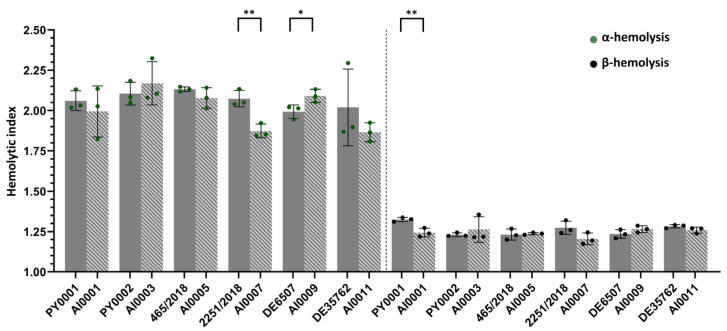
Hemolytic index. Bars show the mean results for the wild type *S. ‘boulardii’* isolates (full grey) and for the *ΔΔHMX1* mutant strains (striped). Individual data points are shown in green (α-hemolysis) and black (β-hemolysis), and whiskers show the standard deviation of the data. Comparisons with significant differences are marked (*: *p* < 0.05; **: *p* < 0.01).

Since it was known that two of the strains were blood isolates (Table 1)—hence considered virulent—on the one hand we intended to consider whether it is evincible by the measurement of α- and β-hemolytic indices that these two isolates are indeed virulent, and on the other hand we wanted to assess the influence of the deletion of *HMX1* on hemolytic activity. When deletion mutant strains were compared to their parental wild-type isolates, significant differences were only found in the case of the non-mycosis isolate 2251/2018 (*p* < 0.01; 2251/2018 > AI0007; α-hemolysis), the mycosis isolate DE6507 (*p* < 0.05, DE6507 < AI0009; α-hemolysis), and the commercial isolate PY0001 (*p* < 0.01, PY0001 > AI0001; β-hemolysis). Hence, strain level comparisons could not reveal a clear tendency towards the effect of *HMX1* loss of function (Figure 5, Supplementary Table S3).

### 3.5. Deletion of HMX1 Caused a Significantly Higher Kidney Burden in Immunosuppressed BALB/c Mice

**Figure 6:**
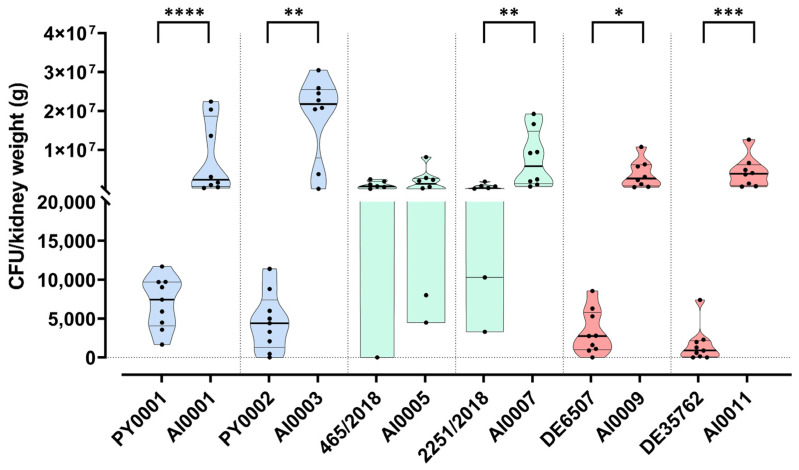
Kidney burden of mice injected with yeast probiotic isolates and *HMX1* deletion mutant strains. Individual data points represent the colony forming units per kidney weight (g) for every mouse. Data from mice that died during or were killed at the end of the experiment are both plotted. Horizontal black lines show the median of the datapoints within a dataset. Comparisons with significant differences are marked (*: *p* < 0.05; **: *p* < 0.01; ***: *p* < 0.001; ****: *p* < 0.0001).

In most studies, the virulence of probiotic yeast is assessed based merely on in vitro experiments; however, this approach can be inaccurate and misleading [10]. Hence, here we put more emphasis on using an animal model to determine the degree of virulence of our isolates and their deletion mutant variants. The yeast isolates and strains were injected into the lateral tail vein of immunosuppressed BALB/c mice, simulating a bloodstream infection. Fungal kidney burden was determined and, except for the strain 465/2018, all the wild-type isolates showed significantly lower CFU values compared to the deletion mutants (Figure 6, Supplementary Table S4). The isolate 465/2018 did not differ significantly from its corresponding deletion mutant AI0005 (Figure 6, Supplementary Table S4). Interestingly, this wild-type isolate caused the highest kidney burden (considering the wild-type isolates) among the mice (Figure 6). Among the mutant strains, AI0003 caused the highest kidney burden (Figure 6).

## 4. Discussion

Several publications describe that probiotic yeasts can cause mycosis and fungemia, creating life-threatening health conditions [9,41,42,71,72]; however, much less data are available on the apparently complex mechanisms [10] and causes of such events. This study aimed to disentangle a part of this issue, focusing on resistance to iron starvation and hemolysis, by inducing the loss of function of a gene with a known virulence factor homologue of *C. albicans*, namely *HMX1*. It plays a central role in the iron metabolism of the yeast *S. cerevisiae* under iron deprivation, which is a common strategy of the human host against invading pathogens [14,15,16,17,18,19]. Hence, we suspected that *HMX1* might play a role in the development of pathogenicity and in the fitness of the yeast reproducing in the host and aimed to test the effect of its deletion in different *S. ’boulardii’* genetic backgrounds. These backgrounds included commercial, mycosis, and non-mycosis isolates that are known to show in-host adaptations and variability in their phenotypes. Using the newly assembled and annotated genome of one of the commercial isolates, we determined that the isolates are indeed closely related, but contain unique mutations and thus represent various genetic backgrounds. While large-scale genome structure variations were not observed, hundreds of mutations affected protein functions in the isolates, and long tracts of heterozygosity losses were occasionally observed. Notable are the 11 genes interacting with *HMX1* with observed high- (two genes) or medium-effect variants (ten genes, one also having a high-effect mutation) that affect protein function, and the fact that numerous mutations were uncovered in various other genes (43 genes having high-effect mutations and 73 showing medium-effect mutations) that differ among the sequenced isolates (Supplementary File S1). This latter, larger group of affected genes highlight how different the genetic backgrounds even among closely related probiotic yeasts may be. These results are reminiscent of the virulence data presented by McCullough et al. [73], who showed, using two batches of the same product, that virulence (defined as growth in the host) may be variable. Such differences in the genetic backgrounds may explain the different outcomes of the iron starvation experiments and the mice infection in this study, detailed below, similarly to how a recent large-scale study showed that variations in *S. cerevisiae* genetic backgrounds substantially affect gain- or loss-of-fitness effects of loss-of-function mutations [74].

After the successful deletion of the gene, we tested the growth under iron starvation by spot plate phenotyping using an iron-deprived medium, and by determining the growth in 50% human serum. The α- and β-hemolytic activities of the isolates and strains were also assessed (Figure 3, Figure 4 and Figure 5; Supplementary Tables S1–S3). Moreover, we conducted animal experiments to determine the growth of the yeasts in a neutropenic mice model (Figure 6; Supplementary Table S4).

Tolerance of iron starvation varied among the strains based on their growth on BPS-supplemented plates and in 50% human serum-50% RPMI-1640 medium. The obtained CFU values in 50% human serum for the deletion strains AI0001, AI0003, and AI0009 were lower than the CFU values for the wild-type isolates (though in the case of AI0009 the difference was not significant). Interestingly, AI0011 showed significantly higher growth compared to DE35762. These results were very similar to the results obtained from the spot plate experiment. The strains AI0005 and AI0007 showed contrasting results since they had decreased growth on the spot plate, but increased growth in serum when compared to the isolates 465/2018 and 2251/2018. In the latter case, the growth difference was not significant. Meanwhile, α- and β-hemolytic activity did not distinguish the strains, despite the fact that it is a commonly applied method for assessing virulence in pathogenic yeasts [34,35,44] and safety in probiotic yeasts [39,40,75]. This observation is notable as hemolysis is unlikely to play a role in the iron metabolism of *S. ’boulardii’*, and hence in its potential virulence.

With mouse infection experiments, we applied a model of bloodstream-derived infection and tested the various yeast isolates and knockout strains, using kidney burden as a proxy to in-host fitness. Our assumption was that if we delete the gene *HMX1*, due to the lack of iron mobilization ability inside the yeast cell, the deletion strains would show lower fitness in the host. Surprisingly, the loss of *HMX1* resulted in a significant increase of CFU values in the case of all strains used in this study, with one exception, AI0005 (Figure 6, Supplementary Table S4). The CFU values were variable especially for isolates 465/2018 and 2251/2018, suggesting that the susceptibility of mice to yeast infection was host individual-dependent as well. However, despite the variability of CFU numbers among mice, and despite the apparent differences in genetic background of the yeasts, in five of the six wild-type vs. knockout comparisons the CFU differences were significantly higher in the strains lacking *HMX1*.

Virulence factors are defined as attributes of the fungal pathogen that cause damage to the host (either by direct action or by the host response) or allow growth and survival (elevating fitness) during infection. These are also called virulence determinants in the latter case [76]. In the case of our gene of interest in this work, in stark contrast to its *C. albicans* homologue [28], *HMX1* in *Saccharomyces* is not a virulence factor gene. The significantly elevated fitness of deletion strains in infected mice in all but one probiotic yeast genetic background is reminiscent of a rarely described phenomenon, the existence of antivirulence genes. These were only recently defined for human pathogenic fungi [76]. The loss or deletion of such genes leads to an increased virulence, either by affecting host responses, or presumably due to compensatory stress responses of the fungal cell that over-compensates the detrimental effect of the gene loss and leads to fitness increase in the stressful host environment.

The data presented in this study are the first evidence of an antivirulence-like gene in *S. ’boulardii’*, suggesting that not exclusively gaining a virulence factor, but also a loss-of-function mutation, might contribute to increased fitness of the probiotic yeast in a mammalian host. Results comparable to this were only observed before in the context of cell wall architecture and higher virulence (the gene *SSD1*), as well as cell wall architecture and higher immune activation in vitro (*MCD4*) in the case of *Saccharomyces* [77,78]. This draws attention to the fact that the deletion of a gene can cause unexpected adaptive benefits potentially promoting pathogenicity. In this study, the mammalian pathogenicity model showed this surprising effect in the form of a significantly higher kideny burden. In vitro experiments, even serum growth tests, did not correlate with these, highlighting the fact that mammal models are insurmountable when testing the potential pathogenic nature of *Saccharomyces* strains. For that reason, genetic engineering and strain development of the *S. ’boulardii’* probiotic yeast should be performed with caution and bearing in mind that it may be a source of health-related problems in the future. In vivo virulence tests of novel probiotic strains, including engineered ones, are therefore highly recommended.

## Figures and Tables

**Table 1:** Commercial and clinical isolates, and their corresponding *ΔΔHMX1* mutant strains used in this study.

ID	Isolate	*ΔΔHMX1* Mutant	Type	Formulation		Component		Place of Acquisition	Date of Acquisition	Country of Manufacturing	Reference
1	PY0001	AI0001	Commercial probiotic	Active dry		Single component		Debrecen, Hungary	March 2015	France	[6]
2	PY0002	AI0003	Commercial probiotic	Active dry		Single component		Debrecen, Hungary	November 2017	France	[6]
**ID**	**Isolate**	***ΔΔHMX1* Mutant**	**Type**	**Age (yr) at Sampling**	**Sex**	**Medical Condition** **during *Saccharomyces* isolation**	**Mycosis Case**	**Anatomical Origin/Sample Type**	**Date of** **Sampling**	**Geographic Origin**	**Reference**
3	465/2018	AI0005	Non-mycosis isolate (probiotic-derived)	41	♀	Amenorrhea	no	Vagina	3 January 2018	Szeged, University Clinic	-
4	2251/2018	AI0007	Non-mycosis isolate (probiotic-derived)	17	♂	Ulcerative colitis	no	Feces	8 January 2018	Szeged, University Clinic	-
5	DE6507	AI0009	Mycosis isolate (probiotic-derived)	63	♂	Pneumonia	yes	Hemoculture	18 February 2017	Debrecen, University Clinic	[6]
6	DE35762	AI0011	Mycosis isolate (probiotic-derived)	66	♀	Respiratory failure	yes	Hemoculture	5 November 2015	Debrecen, University Clinic	[6]

**Table 2:** Oligonucleotide sequences of repair DNA, gRNA, and verification primers. The insertion cassette is indicated with lowercase letters within the repair DNA sequences. gRNA modifications according to Akhmetov et al. [65] are also indicated with lowercase letters.

**Oligos for Repair DNA:**		Forward: 5′-ATGGAGGACAGTAGCAATACAATCATACCCTCACCCACTGACGTGGGGGCGCTAGCAAACcactaactaactaagcgtcg-3′ Reverse: 5′-TTTTGATATTATTTCATGTATATATTATGTTTGTATTTAGACTTTTTTTTTTATACGTTAcgacgcttagttagttagtg-3′
**Oligos for gRNA:**		Forward: 5′-gactttCATCCACGAAAACATACACA-3′ Reverse: 5′-aaacTGTGTATGTTTTCGTGGATGaa-3′
**Verification Primers**	**Primers to Verify Gene Deletion**	Forward: 5′-TGTTATTGTACCCATAGAGG-3′ Reverse: 5′-AAATATAGTGATGCAACTCG-3′
	**Primers to Verify Locus of Insertion**	Forward: 5′-TGTTATTGTACCCATAGAGG-3′ Reverse: 5′-CGACGCTTAGTTAGTTAGTG-3′

## Data Availability

Raw sequencing reads were deposited in SRA (BioProjetcs PRJNA813746, PRJNA813763). The annotated assembly of PY0001 was deposited in GenBank. In FigShare, we deposited the annotated (SNPEff) VCF and the gVCF files, along with photographs of Petri dishes.

## Supplementary Materials

The following supporting information can be downloaded at: https://www.mdpi.com/article/10.3390/jof8050522/s1, Supplementary File S1, Comparative genomic results of the probiotic isolates used in this study. Supplementary Tables S1–S4, Supplementary Figure S1.
