# Supplementary material for: Heme Oxygenase-1 (HMX1) Loss of Function Increases the In-Host Fitness of the Saccharomyces ‘boulardii’ Probiotic Yeast in a Mouse Fungemia Model
Source: J Fungi (Basel). 2022 May 18;8(5):522. doi: 10.3390/jof8050522 (PMC9146039; doi:10.3390/jof8050522)
Supplement: Supplementary file 1 [file jof-08-00522-s001.zip › Suplementary_figure_S1.pdf]

Supplementary Figure S1.

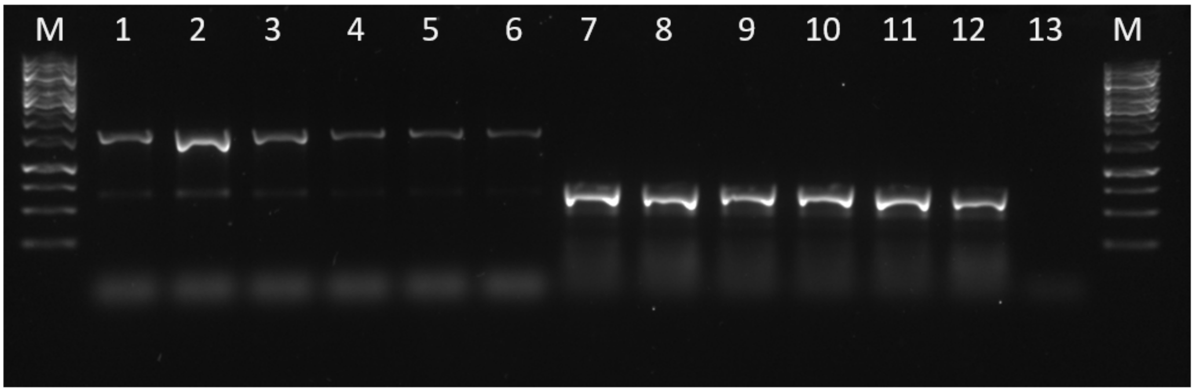

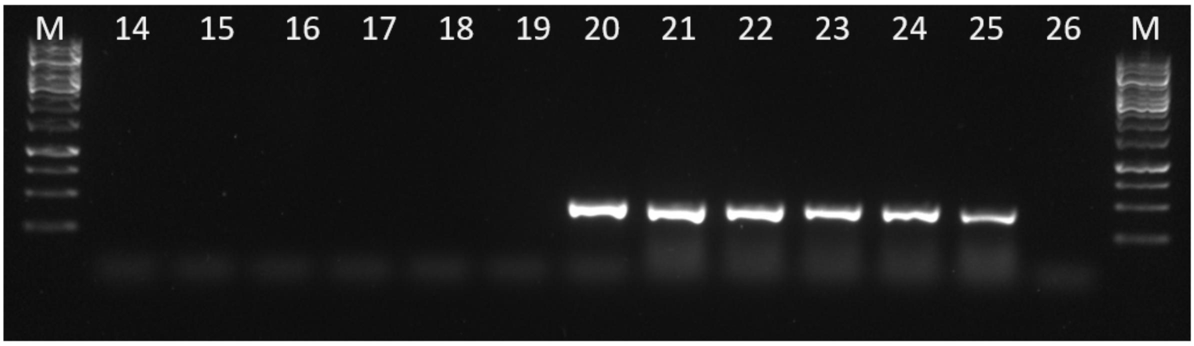

PCR verification of CRISPR/Cas9 induced *HMX1* gene deletion. The upper image shows the products resulting from PCR amplification of the *HMX1* ORF in wild type (1: PY0001; 2: PY0002; 3: 465/2018; 4: 2251/2018; 5: DE6507; 6: DE35762) and mutant strains (7: AI0001; 8: AI0003; 9: AI0005; 10: AI0007; 11: AI0009; 12: AI0011). The lower image shows the products (or the lack of products) resulting from PCR amplification of the repair cassette in wild type (14: PY0001; 15: PY0002; 16: 465/2018; 17: 2251/2018; 18: DE6507; 19: DE35762) and mutant strains (20: AI0001; 21: AI0003; 22: AI0005; 23: AI0007; 24: AI0009; 25: AI0011). Sample 13 and 26 were the negative controls (master mix without genomic DNA) for the applied primer mix 1 and primer mix 2, respectively. M: 1 kb DNA ladder (Thermo Fisher).
