# Supplementary material for: Heme Oxygenase-1 (HMX1) Loss of Function Increases the In-Host Fitness of the Saccharomyces ‘boulardii’ Probiotic Yeast in a Mouse Fungemia Model
Source: J Fungi (Basel). 2022 May 18;8(5):522. doi: 10.3390/jof8050522 (PMC9146039; doi:10.3390/jof8050522)
Supplement: Supplementary file 1 [file jof-08-00522-s001.zip › Supplementary_file_S1.pdf]

Supplementary file S1.

Comparative genomic results of the probiotic isolates used in this study.

Features of the assembled genome of the PY0001 isolate: length of chromosomes compared with the *S. cerevisiae* S288c reference genome, and the number of the annotated protein coding genes. The mitochondrial genome of the strain CIM was used as it was not assembled for PY0001.

Chromosome	S288c reference genome	PY0001 reference (this study)
**Chr. I.**	230218 bp	210 734 bp, 95 genes
**Chr. II.**	813 184 bp	793 175 bp, 397 genes
**Chr. III.**	316 620 bp	315 973 bp, 159 genes
**Chr. IV.**	1 531 933 bp	1 510 005 bp, 743 genes
**Chr. V.**	576 874 bp	565 474 bp, 273 genes
**Chr. VI.**	270 161 bp	245 370 bp, 122 genes
**Chr. VII.**	1 090 940 bp	1 058 692 bp, 503 genes
**Chr. VIII.**	562 643 bp	523 461 bp, 271 genes
**Chr. IX.**	439 888 bp	420 152 bp, 208 genes
**Chr. X.**	745 751 bp	730 649 bp, 354 genes
**Chr. XI.**	666 816 bp	668 834 bp, 321 genes
**Chr. XII.**	1 078 177 bp	1 051 807 bp, 472 genes
**Chr. XIII.**	924 431 bp	909 758 bp, 450 genes
**Chr. XIV.**	784 333 bp	790 714 bp, 382 genes
**Chr. XV.**	1 091 291 bp	1 054 797 bp, 511 genes
**Chr. XVI.**	948 066 bp	931 421 bp, 456 genes
**mtDNA**	85 779 bp	87 321 bp, 20 genes
**plasmid**	none	not assembled

Whole genome alignment of the S288c reference *S. cerevisae* and PY0001 reference genomes. Chromosomes are shown with grey bars on the axes.

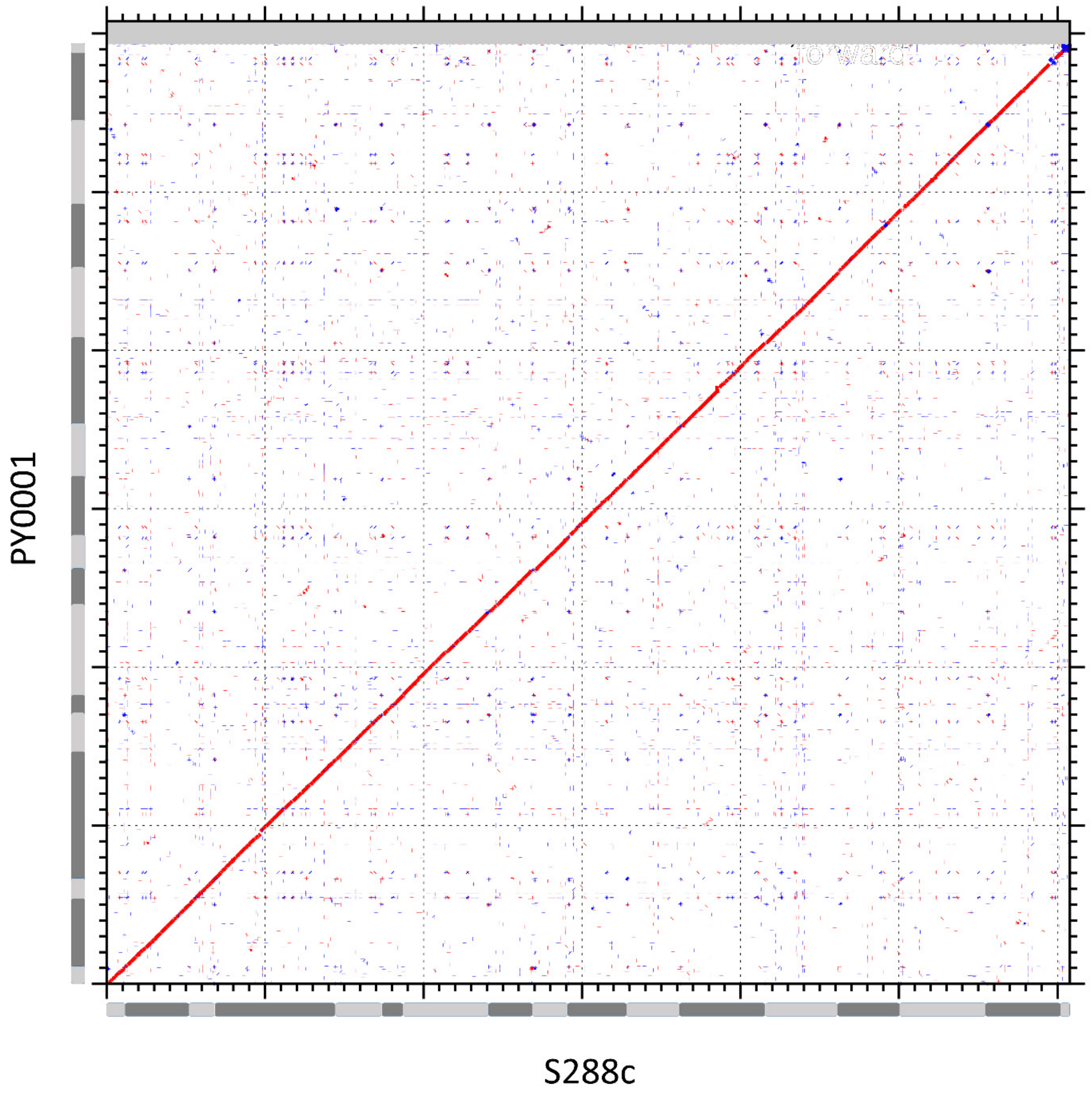

Phylogenetic relationships of the 6 strains used in this study. a) Maximum Likelihood dendrogram based on the identified polymorphisms. Support for branches with a higher than 80% bootstrap approximation support is indicated. ADD represents the Y-prime amplification subclade of the Win/European clade, and it is used as an outgroup. b) Network based on whole genome SNPs and considering heterozygous positions (by averaging the uncorrected P distance of the two haplotypes).

a.

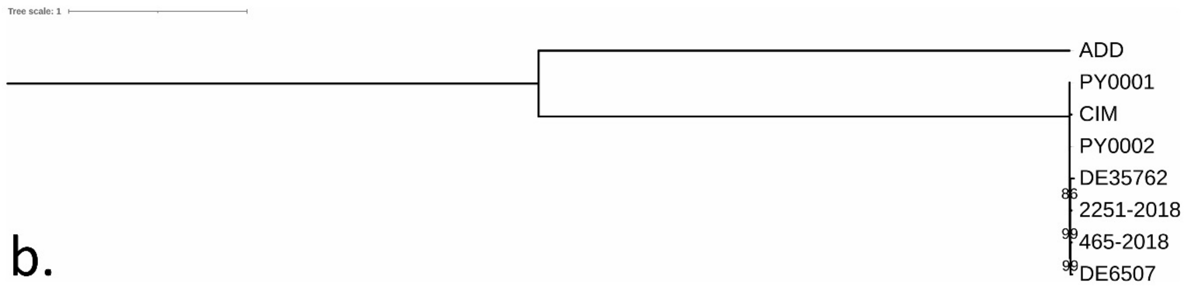

b.

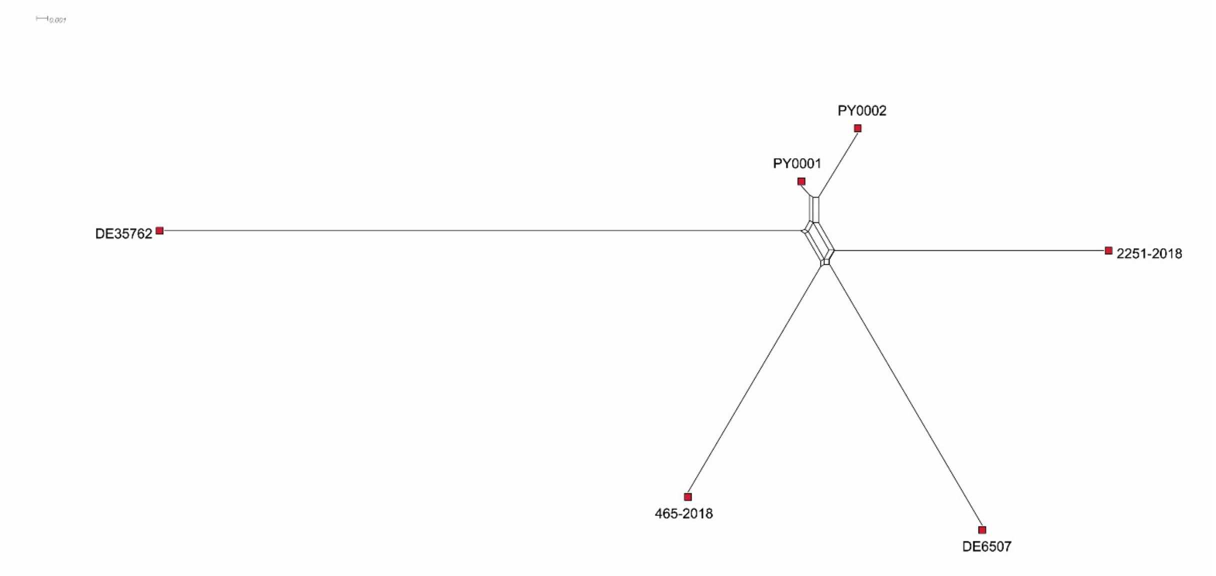

Coverage data for each sequenced isolate with 10 000 bp sliding windows (sliding every 5 000 bp). Disomy can be observed for all 16 chromosomes, and the peak caused by ribosomal DNA repeats can be seen on chr. XII. Mitochondrial coverage is higher due to high copy number. The x-axis shows the chromosomes and the y-axis shows the number of copies calculated from coverage.

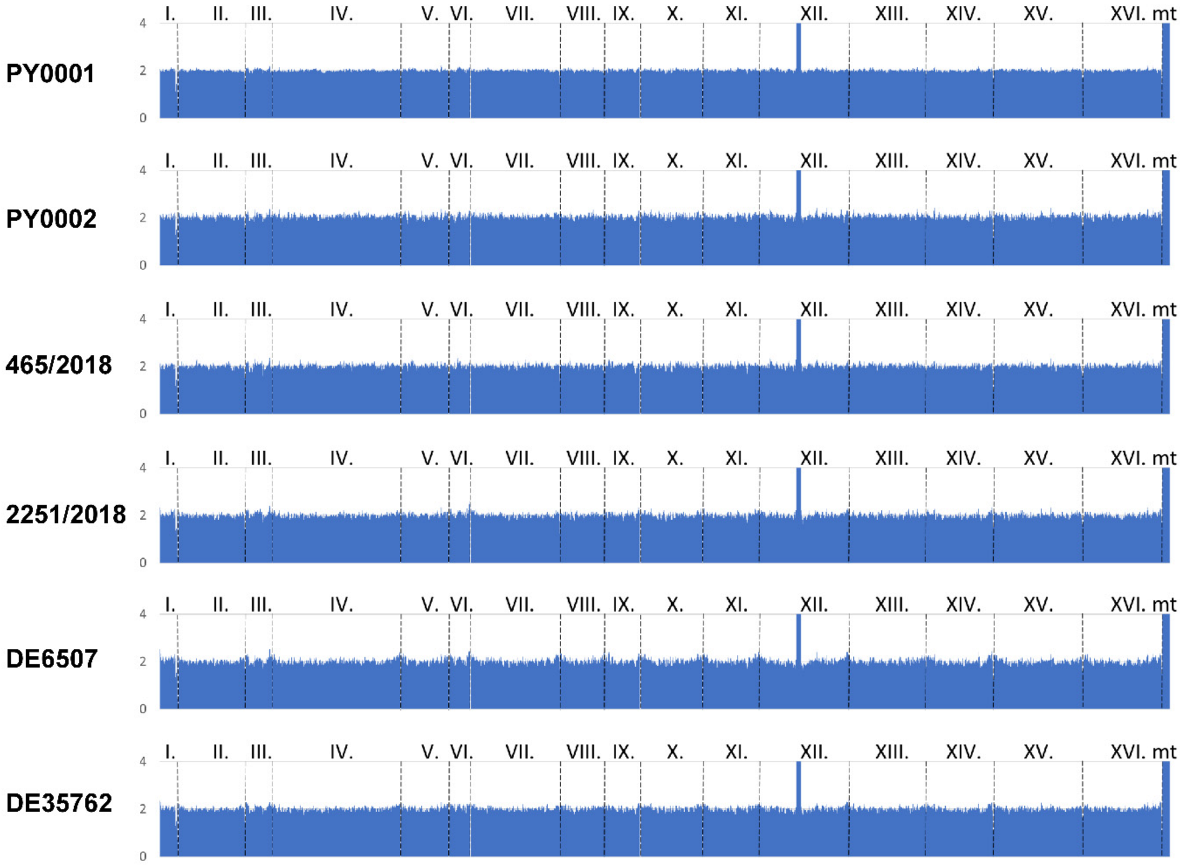

Differences in gene copy numbers observed between isolates. Copy number differences were identified in only 10 genes on 8 different chromosomes based on coverage data. Copy numbers are ranging from 1 to 2 per haploid genome.

Gene name	Description	PY0001	PY0002	465/2018	2251/2018	DE6507	DE35762
G0000950	dubious telomeric	1	1	1	2	2	1
YBR196C-A	uncharacterized	1	1	1	2	1	1
YER188C-A	uncharacterized	2	1	2	2	2	2
YDL069C	mitochondrial respiratory chain complex III assembly	1	2	1	1	1	1
YDL009C	uncharacterized	1	2	1	1	1	1
G0021860	dubious	1	1	1	2	1	1
G0025660	dubious	1	1	1	1	2	2
YLR211C	involved in macroautophagy	1	1	1	1	2	1
G0039200	dubious telomeric	1	1	1	2	1	1
YPR166C	involved in mitochondrial translation	1	2	1	1	1	1

Copy number differences between sequenced isolates in mitochondrial and plasmid copy number, and copy numbers of rDNA elements and retrotransposons relative to haploid genome. Isolates are color-coded on the bar chart.

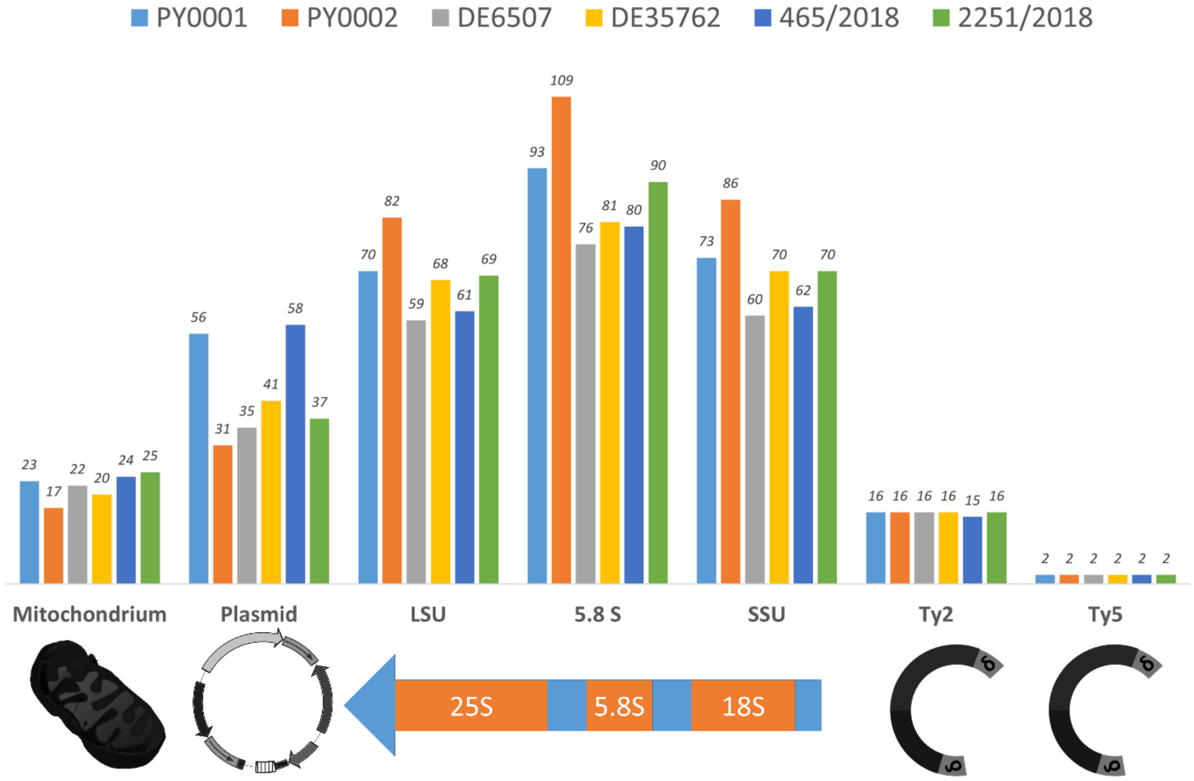

Ratio of AD values (y-axis) of variants (insertion / deletion and SNP variants) identified on the chromosomes (x-axis) of the sequenced isolates. Each data point shows the AD ratios of individual variants. The difference in the number of heterozygous variants on each chromosome is notable.

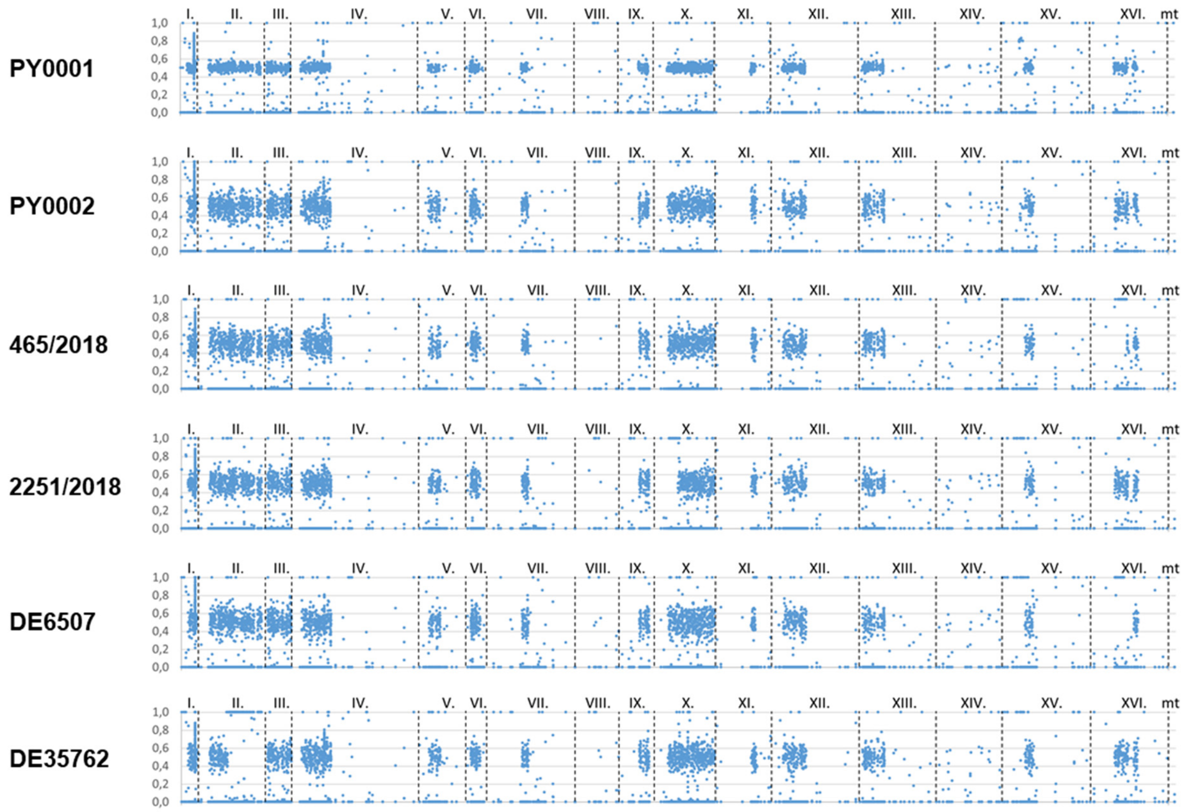

Heterozygosity (percentage of heterozygous nucleotides) along each chromosome in the six sequenced isolates.

	PY0001	PY0002	465/2018	2251/2018	DE6507	DE35762
Chr. I.	0.14810%	0.13670%	0.14280%	0.14140%	0.13810%	0.14620%
Chr. II.	0.05420%	0.05360%	0.05410%	0.05300%	0.05400%	0.02030%
Chr. III.	0.07150%	0.07180%	0.07030%	0.06740%	0.07180%	0.07090%
Chr. IV.	0.02020%	0.01980%	0.02070%	0.02000%	0.02050%	0.02050%
Chr. V.	0.01500%	0.01540%	0.01490%	0.01470%	0.01520%	0.01500%
Chr. VI.	0.04690%	0.04690%	0.04120%	0.04650%	0.04650%	0.04610%
Chr. VII.	0.00760%	0.00740%	0.00720%	0.00700%	0.00710%	0.00720%
Chr. VIII.	0.00060%	0.00020%	0.00040%	0.00060%	0.00110%	0.00080%
Chr. IX.	0.02280%	0.02170%	0.02140%	0.02190%	0.02170%	0.02120%
Chr. X.	0.05980%	0.05870%	0.05940%	0.04780%	0.05980%	0.06010%
Chr. XI.	0.00990%	0.00960%	0.00960%	0.00990%	0.00750%	0.01000%
Chr. XII.	0.01630%	0.01570%	0.01610%	0.01630%	0.01600%	0.01610%
Chr. XIII.	0.01580%	0.01490%	0.01550%	0.01590%	0.01570%	0.01600%
Chr. XIV.	0.00240%	0.00230%	0.00230%	0.00270%	0.00240%	0.00230%
Chr. XV.	0.00780%	0.00700%	0.00650%	0.00640%	0.00610%	0.00670%
Chr. XVI.	0.01670%	0.01630%	0.00700%	0.01600%	0.00460%	0.01650%

Locations of longer (minimum 10 variant positions) tracts of heterozygosity losses and gains when the sequence isolates are compared to PY0001.

Chromosome	start	end	PY0002	465/2018	2251/2018	DE6507	DE35762
**chr. I.**	169 499	169 588	loss				
**chr. II.**	349 630	750 382					loss
**chr. IV.**	390 373	390 430		gain		gain	gain
**chr. VI.**	125 779	128 246		loss			
**chr. X.**	84 809	284 582			loss		
**chr. XI.**	478 095	486 704				loss	
**chr. XVI.**	290 756	425 832		loss			
**chr. XVI.**	290 756	489 943				loss	

List of genes affected by high effect mutations in the genomes of each isolate, including descripton of the genes. Gene names beginning with 'G' are dubious ORFs found during annotation, without determined homologies.

**List of affected strains**	**Affected genes**	**Description of affected genes**
**PY0002, 465/2018, 2251/2018, DE6507, DE35762**	*G0005430*	not characterized during annotation
	G0005510	not characterized during annotation
	G0005990	not characterized during annotation
	G0008480	not characterized during annotation
	G0008490	not characterized during annotation
	G0008580	not characterized during annotation
	G0008590	not characterized during annotation
	G0008670	not characterized during annotation
	G0027120	not characterized during annotation
	G0028660	not characterized during annotation
	G0028710	not characterized during annotation
	G0028750	not characterized during annotation
	G0028760	not characterized during annotation
	G0029600	not characterized during annotation
	YCL014W	Guanine nucleotide exchange factor (GEF) for Cdc42p
	YCL024W	Protein kinase of the bud neck involved in the septin checkpoint
	YCR014C	DNA polymerase IV
	YDL003W	Essential alpha-kleisin subunit of the cohesin complex
	YDL009C	Protein of unknown function
	YDL013W	Subunit of the Slx5-Slx8 SUMO-targeted Ub ligase (STUbL) complex
	YDL030W	Subunit of the SF3a splicing factor complex
	YEL016C	Nucleotide pyrophosphatase/phosphodiesterase
	YJL002C	Alpha subunit of the oligosaccharyltransferase complex of the ER lumen
	YJL005W	Adenylate cyclase
	YJL023C	Protein of unknown function
	YJL070C	Putative metallo-dependent hydrolase superfamily protein
	YJL071W	Acetylglutamate synthase (glutamate N-acetyltransferase)
	YJL076W	Core subunit of the RENT complex
	YJR050W	Member of the NineTeen Complex (NTC)
	YJR053W	Subunit of a two-component GTPase-activating protein, Bfa1p-Bub2p
	YPL105C	Protein of unknown function that influences nuclear pore distribution

	YPL116W	Trichostatin A-insensitive homodimeric histone deacetylase (HDAC)
	YPL119C-A	Putative protein of unknown function
**PY0001, PY0002, 465/2018, 2251/2018, DE6507**	*YBR132C*	Plasma membrane regulator of polyamine and carnitine transport
	YER083C	Subunit of the GET complex
**PY0001, PY0002, 2251/2018, DE35762, DE6507**	*YDL039C*	Pheromone-regulated protein
	YFR028C	Protein phosphatase required for mitotic exit
**PY0001, 2251/2018, DE35762**	*G0029090*	dubious/partial
**PY0001, 2251/2018**	*YOR305W*	Protein of unknown function
**2251/2018**	*YEL013W*	Vacuole-specific Myo2p receptor
**DE6507**	*YHR080C*	Sterol-binding protein that localizes to puncta in the cortical ER
	YMR127C	Histone acetyltransferase (HAT) catalytic subunit of the SAS complex
**DE35762**	*YKR101W*	Protein involved in silencing at mating-type loci HML and HMR
**All 6 isolates**	*G0000760*	not characterized during annotation
	G0002090	not characterized during annotation
	G0002480	not characterized during annotation
	G0006270	not characterized during annotation
	G0008130	not characterized during annotation
	G0008160	not characterized during annotation
	G0008510	not characterized during annotation
	G0016810	not characterized during annotation
	G0017310	not characterized during annotation
	G0017400	not characterized during annotation
	G0029090	not characterized during annotation
	G0029890	not characterized during annotation
	G0031090	not characterized during annotation
	G0034190	not characterized during annotation
	G0036200	not characterized during annotation
	G0040610	not characterized during annotation
	G0048920	not characterized during annotation
	YAR027W	Putative integral membrane protein of unknown function
	YBR058C	Ubiquitin-specific protease
	YBR148W	Protein required for normal prospore membrane formation
	YBR260C	GTPase-activating protein (RhoGAP) for Rho3p and Rho4p
	YCL049C	Protein of unknown function

	YCL051W	Protein involved in control of cell wall structure and stress response
	YCR091W	Putative serine/threonine protein kinase
	YCR100C	Protein involved in protein sorting
	YCR106W	Putative zinc cluster transcription factor
	YCL049C	Protein of unknown function
	YDL109C	Putative lipase
	YDL171C	NAD(+)-dependent glutamate synthase (GOGAT)
	YER093C	Subunit of TORC2 (Tor2p-Lst8p-Avo1-Avo2-Tsc11p-Bit61p)
	YFL018C	Dihydrolipoamide dehydrogenase
	YFR047C	Quinolinate phosphoribosyl transferase
	YIR003W	Subunit of a complex that associates with actin filaments
	YJL050W	RNA duplex-sensing translocase
	YJR040W	Voltage-gated chloride channel
	YJR131W	Alpha-1,2-mannosidase
	YJR134C	Protein of unknown function
	YJR148W	Cytosolic branched-chain amino acid (BCAA) aminotransferase
	YKR002W	Poly(A) polymerase
	YLR006C	Cytoplasmic phosphorelay intermediate osmosensor and regulator
	YLR078C	v-SNARE (vesicle specific SNAP receptor)
	YLR145W	Subunit of RNase MRP
	YNL113W	RNA polymerase subunit AC19
	YNL317W	Integral subunit of the pre-mRNA CPF complex
	YOR032C	bHLH protein with similarity to myc-family transcription factors
	YOR037W	Mitochondrial peripheral inner membrane protein
	YOR043W	Negative regulator of TORC1 in response to limiting leucine

List of genes affected by moderate effect mutations in the genomes of each isolate, including description of the genes.

List of affected strains	Affected genes	Description of affected genes
**PY0001, 465/2018, 2251/2018, DE6507, DE35762**	*YLR086W*	Subunit of the condensin complex
**PY0001, PY0002, 2251/2018, DE6507, DE35762**	*YFR003C*	Regulatory subunit of the type I protein phosphatase (PP1) Glc7p
	YPL061W	Cytosolic aldehyde dehydrogenase
	YPL064C	Component of a complex containing Cef1p
	YPL065W	Component of the ESCRT-I complex
	YPL085W	COPII vesicle coat protein required for ER transport vesicle budding
	YPL101W	Subunit of hexameric RecA-like ATPase Elp456 Elongator subcomplex
	YPL104W	Mitochondrial aspartyl-tRNA synthetase
	YPL105C	Protein of unknown function that influences nuclear pore distribution
	YPL106C	ATPase component of heat shock protein Hsp90 chaperone complex
	YPL108W	Cytoplasmic protein of unknown function
	YPL110C	Glycerophosphocholine (GroPCho) phosphodiesterase
	YPL113C	Glyoxylate reductase
**PY0001, PY0002, 465/2018, DE6507, DE35762**	*G0028750*	not characterized during annotation
	G0028770	not characterized during annotation
	YBR036C	Endoplasmic reticulum membrane protein
	YBR037C	Copper-binding protein of mitochondrial inner membrane
	YCR045C	Probable subtilisin-family protease
	YJL092W	DNA helicase and DNA-dependent ATPase
	YJL106W	Serine/threonine protein kinase involved in activation of meiosis
	YJL111W	Subunit of the cytosolic chaperonin Cct ring complex
	YJL115W	Nucleosome assembly factor
	YJL123C	Protein of unknown function that may interact with ribosomes
**PY0001, PY0002, 465/2018, 2251/2018, DE35762**	*YKR023W*	Subunit of ribosome-associated quality control trigger complex (RQT)
	YKR024C	Putative ATP-dependent RNA helicase of the DEAD-box family
	YPL083C	Subunit of the tRNA splicing endonuclease

	YPL084W	Cytoplasmic class E vacuolar protein sorting (VPS) factor
	YPL126W	U3 snoRNP protein
	YPL130W	Meiosis-specific prospore protein
	YPL133C	Transcription factor involved in regulating gluconeogenesis
**PY0001, PY0002, 465/2018, 2251/2018, DE6507**	*G0004120*	not characterized during annotation
	YBR078W	GPI-anchored protein involved in efficient glucose uptake
	YBR081C	Subunit of the SAGA transcriptional regulatory complex
	YBR084W	Mitochondrial C1-tetrahydrofolate synthase
	YBR094W	Putative tubulin tyrosine ligase associated with P-bodies
	YBR097W	Serine/threonine protein kinase involved in vacuolar protein sorting
	YBR098W	Subunit of structure-specific Mms4p-Mus81p endonuclease
	YBR103W	WD40 repeat-containing subunit of Set3C histone deacetylase complex
	YBR115C	Alpha aminoadipate reductase
	YBR122C	Mitochondrial ribosomal protein of the large subunit
	YBR136W	Genome integrity checkpoint protein and PI kinase superfamily member
	YBR141C	Nucleolar S-adenosylmethionine-dependent rRNA methyltransferase
	YBR156C	Subunit of the conserved chromosomal passenger complex (CPC)
	YBR222C	Oxalyl-CoA synthetase
	YBR225W	Putative protein of unknown function
	YBR260C	GTPase-activating protein (RhoGAP) for Rho3p and Rho4p
	YBR272C	Evolutionarily conserved 19S regulatory particle assembly-chaperone
**PY0001, PY0002, DE35762**	*G0049070*	not characterized during annotation
**PY0001, 2251/2018, DE35762**	*G0029600*	not characterized during annotation
**PY0001, PY0002**	*YOL016C*	Calmodulin-dependent protein kinase
	YOL023W	Mitochondrial translation initiation factor 2
	YOL025W	Protein that negatively regulates the SCF E3-ubiquitin ligase
	YOL036W	Protein of unknown function
	YOL040C	Protein component of the small (40S) ribosomal subunit
	YOL044W	Tail-anchored type II integral peroxisomal membrane protein
**PY0001**	*YDL014W*	Histone glutamine methyltransferase
**PY0002**	*G0047510*	not characterized during annotation
**2251/2018**	*G0039660*	not characterized during annotation

	YDR101C	Nuclear export factor for the ribosomal pre-60S subunit
	YDR513W	Cytoplasmic glutaredoxin
**DE6507**	*YGL113W*	Protein involved in the initiation of DNA replication
	YHR074W	Glutamine-dependent NAD(+) synthetase
	YKL062W	Stress-responsive transcriptional activator
**DE35762**	*G0029090*	not characterized during annotation
	YHR098C	Component of the Sec23p-Sfb3p heterodimer of the COPII vesicle coat
	YIL088C	Vacuolar amino acid transporter
	YJR111C	Peroxisomal matrix protein with naturally active promoter
	YKR101W	Protein involved in silencing at mating-type loci HML and HMR
	YLR383W	Subunit of the SMC5-SMC6 complex
	YLR398C	Ski complex component and putative RNA helicase
	YNL055C	Mitochondrial porin (voltage-dependent anion channel)
	YOR307C	Protein involved in ER-to-Golgi transport
**All 6 isolates**	*G0000750*	not characterized during annotation
	G0000760	not characterized during annotation
	G0000770	not characterized during annotation
	G0000800	not characterized during annotation
	G0001840	not characterized during annotation
	G0002090	not characterized during annotation
	G0005430	not characterized during annotation
	G0005450	not characterized during annotation
	G0006190	not characterized during annotation
	G0006260	not characterized during annotation
	G0007310	not characterized during annotation
	G0007670	not characterized during annotation
	G0008160	not characterized during annotation
	G0008510	not characterized during annotation
	G0008670	not characterized during annotation
	G0020230	not characterized during annotation
	G0026820	not characterized during annotation
	G0029590	not characterized during annotation
	G0029930	not characterized during annotation
	G0035540	not characterized during annotation
	G0049200	not characterized during annotation
	G0049350	not characterized during annotation
	YAL007C	Member of the p24 family involved in ER to Golgi transport
	YAL008W	Integral mitochondrial outer membrane (MOM) protein
	YAL010C	Subunit of both the ERMES and the SAM complex
	YAL011W	Protein of unknown function
	YAL013W	Component of the Rpd3L histone deacetylase complex
	YAL014C	Endosomal SNARE related to mammalian syntaxin 8

	YAL018C	Protein Involved in spore wall assembly
	YAL019W	Snf2p family member with ATP-dependent chromatin remodeling activity
	YAL021C	Component of the CCR4-NOT transcriptional complex
	YAR002W	FG-nucleoporin component of central core of the nuclear pore complex
	YAR003W	Subunit of the COMPASS (Set1C) complex
	YAR007C	Subunit of heterotrimeric Replication Protein A (RPA)
	YAR014C	Protein involved in bud-site selection
	YAR018C	Nonessential serine/threonine protein kinase
	YAR027W	Putative integral membrane protein of unknown function
	YAR042W	Protein similar to mammalian oxysterol-binding protein
	YBL007C	Cytoskeletal protein binding protein
	YBL008W	Subunit of the HIR complex
	YBL009W	Atypical protein kinase
	YBL010C	Clathrin adaptor (adaptin) accessory protein
	YBL014C	Component of the core factor (CF) rDNA transcription factor complex
	YBL017C	Type I transmembrane sorting receptor for multiple vacuolar hydrolases
	YBL019W	Class II abasic (AP) endonuclease involved in repair of DNA damage
	YBL022C	ATP-dependent Lon protease
	YBL023C	Protein involved in DNA replication
	YBL024W	S-adenosyl-L-methionine-dependent tRNA: m5C-methyltransferase
	YBL032W	RNA binding protein involved in asymmetric localization of ASH1 mRNA
	YBL033C	GTP cyclohydrolase II
	YBL034C	Microtubule plus-end-tracking non-motor protein
	YBL035C	B subunit of DNA polymerase alpha-primase complex
	YBR001C	Putative neutral trehalase, required for thermotolerance
	YBR015C	Alpha-1,2-mannosyltransferase
	YBR020W	Galactokinase
	YBR021W	Plasma membrane localized uracil permease
	YBR023C	Chitin synthase III
	YBR033W	Putative zinc cluster protein, predicted to be a transcription factor
	YBR040W	Integral membrane protein required for efficient mating
	YBR043C	Multidrug transporter of the major facilitator superfamily
	YBR049C	RNA polymerase I enhancer binding protein
	YBR050C	Regulatory subunit of the Glc7p type-1 protein phosphatase
	YBR052C	Protein of unknown function
	YBR053C	Putative protein of unknown function
	YBR058C	Ubiquitin-specific protease

	YBR059C	Ser/Thr protein kinase
	YBR065C	Pre-mRNA splicing factor
	YBR074W	Multi-spanning vacuolar membrane protease
	YBR079C	eIF3a subunit of the eukaryotic translation initiation factor 3 (eIF3)
	YBR080C	AAA ATPase and SNARE disassembly chaperone
	YBR086C	Cortical ER protein involved in ER-plasma membrane (PM) tethering
	YBR093C	Repressible acid phosphatase
	YBR101C	Hsp70 (Ssa1p) nucleotide exchange factor
	YBR102C	Exocyst subunit with dual roles in exocytosis and spliceosome assembly
	YBR112C	General transcriptional co-repressor
	YBR138C	Cytoplasmic protein of unknown function
	YBR140C	GTPase-activating protein
	YBR148W	Protein required for normal prospore membrane formation
	YBR150C	Protein of unknown function
	YBR152W	mRNA splicing factor, component of U4/U6.U5 tri-snRNP
	YBR153W	Diaminohydroxyphoshoribosylaminopyrimidine deaminase
	YBR163W	Mitochondrial 5'-3' exonuclease and sliding exonuclease
	YBR169C	Member of Hsp110 subclass of the heat shock protein 70 (HSP70) family
	YBR172C	GYF domain protein
	YBR175W	Essential subunit of the COMPASS (Set1C) complex
	YBR176W	Ketopantoate hydroxymethyltransferase
	YBR180W	Putative dityrosine transporter of the major facilitator superfamily
	YBR182C	MADS-box transcription factor involved in osmotic stress response
	YBR186W	Hexameric ring ATPase that remodels chromosome axis protein Hop1p
	YBR187W	Calcium and manganese transporter with higher affinity for Ca2+
	YBR196C	Glycolytic enzyme phosphoglucose isomerase
	YBR196C-A	Putative protein of unknown function
	YBR199W	Glycosyltransferase involved in protein glycosylation
	YBR204C	Serine hydrolase
	YBR207W	Putative high affinity iron transporter
	YBR208C	Urea amidolyase
	YBR229C	Glucosidase II catalytic subunit
	YBR253W	Subunit of the RNA polymerase II mediator complex
	YBR255W	Protein of unknown function
	YBR267W	Cytoplasmic pre-60S factor
	YBR270C	Subunit of TORC2 membrane-associated complex

	YBR271W	S-adenosylmethionine-dependent methyltransferase
	YBR274W	Serine/threonine kinase and DNA damage checkpoint effector
	YBR275C	Protein involved in control of DNA replication
	YBR280C	F-Box protein involved in proteasome-dependent degradation of Aah1p
	YBR281C	Component of glutamine amidotransferase (GATase II)
	YCL004W	Phosphatidylglycerolphosphate synthase
	YCL014W	Guanine nucleotide exchange factor (GEF) for Cdc42p
	YCL018W	Beta-isopropylmalate dehydrogenase (IMDH)
	YCL024W	Protein kinase of the bud neck involved in the septin checkpoint
	YCL025C	Broad-specificity, low-affinity amino acid permease
	YCL027W	Membrane protein localized to the shmoo tip
	YCL029C	Microtubule-associated protein
	YCL030C	Multifunctional enzyme containing phosphoribosyl-ATP pyrophosphatase
	YCL036W	Protein of unknown function
	YCL039W	Subunit of GID Complex that binds directly to central component Vid30p
	YCL043C	Protein disulfide isomerase
	YCL048W-A	Putative protein of unknown function
	YCL052C	Component of glycosylphosphatidylinositol-mannosyltransferase I
	YCL054W	AdoMet-dependent methyltransferase
	YCR003W	Mitochondrial ribosomal protein of the large subunit
	YCR011C	Putative ATP-dependent permease of the ABC transporter family
	YCR014C	DNA polymerase IV
	YCR015C	Protein required for cold tolerance
	YCR017C	GPI lipid remodelase
	YCR032W	Protein homologous to Chediak-Higashi syndrome and Beige proteins
	YCR042C	TFIID subunit (150 kDa)
	YCR047C	Ribosome biogenesis factor
	YCR059C	Negative regulator of eIF2 kinase Gcn2p
	YCR065W	Forkhead transcription factor
	YCR075C	Protein involved in cystine transport
	YCR077C	Deadenylation-dependent mRNA-decapping factor
	YCR079W	Mitochondrial type 2C protein phosphatase (PP2C)
	YCR083W	Mitochondrial thioredoxin
	YCR089W	Cell wall adhesin, expressed specifically during mating
	YCR092C	Mismatch repair protein
	YCR093W	Subunit of the CCR4-NOT1 core complex
	YCR106W	Putative zinc cluster transcription factor

	YDL010W	Cis-Golgi localized monothiol glutaredoxin, binds Fe-S cluster
	YDL015C	Enoyl reductase
	YDL019C	Member of an oxysterol-binding protein family with seven members
	YDL024C	Protein of unknown function
	YDL025C	Putative protein kinase, potentially phosphorylated by Cdc28p
	YDL030W	Subunit of the SF3a splicing factor complex
	YDL033C	tRNA-specific 2-thiouridylase
	YDL035C	Plasma membrane G protein coupled receptor (GPCR)
	YDL036C	Mitochondrial tRNA:pseudouridine synthase
	YDL039C	Pheromone-regulated protein
	YDL044C	Mitochondrial protein that interacts with mitochondrial RNA polymerase
	YDL049C	Protein with similarity to Kre9p
	YDL052C	1-acyl-sn-glycerol-3-phosphate acyltransferase
	YDL055C	GDP-mannose pyrophosphorylase (mannose-1-phosphate guanyltransferase)
	YDL056W	Transcription factor
	YDL058W	Essential protein involved in vesicle-mediated ER to Golgi transport
	YDL065C	Chaperone and import receptor for newly-synthesized class I PMPs
	YDL070W	Protein involved in transcription initiation
	YDL073W	Scaffold protein in the HKR1 sub-branch of the Hog1p-signaling pathway
	YDL074C	E3 ubiquitin ligase
	YDL086W	Putative carboxymethylenebutenolidase
	YDL089W	Protein involved in regulation of mitotic exit
	YDL095W	Protein O-mannosyltransferase of the ER membrane
	YDL097C	Essential, non-ATPase regulatory subunit of the 26S proteasome lid
	YDL098C	Component of the U4/U6.U5 snRNP complex
	YDL099W	Cis-golgi localized protein involved in ER to Golgi transport
	YDL100C	Guanine nucleotide exchange factor for Gpa1p
	YDL102W	Catalytic subunit of DNA polymerase delta
	YDL104C	Protein involved in threonylcarbamoyl adenosine biosynthesis
	YDL106C	Homeobox transcription factor
	YDL109C	Putative lipase
	YDL113C	Sorting nexin family member
	YDL117W	SH3-domain protein located in the bud neck and cytokinetic actin ring
	YDL123W	Protein of unknown function

	YDL124W	NADPH-dependent alpha-keto amide reductase
	YDL134C	Catalytic subunit of protein phosphatase 2A, PP2A
	YDL136W	Ribosomal 60S subunit protein L35B
	YDL138W	Plasma membrane high glucose sensor that regulates glucose transport
	YDL139C	Nonhistone component of centromeric chromatin
	YDL140C	RNA polymerase II largest subunit B220
	YDL143W	Subunit of the cytosolic chaperonin Cct ring complex
	YDL145C	Alpha subunit of COPI vesicle coatomer complex
	YDL149W	Transmembrane protein involved in forming Cvt and autophagic vesicles
	YDL154W	Protein of the MutS family
	YDL155W	B-type cyclin involved in cell cycle progression
	YDL171C	NAD(+)-dependent glutamate synthase (GOGAT)
	YDL174C	Major mitochondrial D-lactate dehydrogenase
	YDL186W	Putative protein of unknown function
	YDL193W	Forms dehydrodolichyl diphosphate synthase complex with RER2 or SRT1
	YDR005C	Highly conserved negative regulator of RNA polymerase III
	YDR006C	Protein of unknown function
	YDR009W	Transcriptional regulator
	YDR390C	Subunit of heterodimeric nuclear SUMO activating enzyme E1 with Aos1p
	YEL007W	Transcriptional regulator of pseudohyphal growth
	YEL011W	Glycogen branching enzyme, involved in glycogen accumulation
	YER004W	Protein of unknown function
	YER007W	Microtubule effector required for tubulin heterodimer formation
	YER008C	Subunit of the exocyst complex
	YER022W	Subunit of the RNA polymerase II mediator complex
	YER033C	Protein of unknown function
	YER034W	Protein of unknown function
	YER039C	Protein of unknown function
	YER040W	Transcriptional activator in nitrogen catabolite repression system
	YER041W	Holliday junction resolvase
	YER042W	Methionine-S-sulfoxide reductase
	YER044C-A	Meiosis-specific protein involved in forming DSBs
	YER045C	ATF/CREB family basic leucine zipper (bZIP) transcription factor
	YER047C	Putative ATPase of the AAA family
	YER050C	Mitochondrial ribosomal protein of the small subunit
	YER051W	JmjC domain family histone demethylase specific for H3-K36

	YER054C	Putative regulatory subunit of protein phosphatase Glc7p
	YFL001W	tRNA:pseudouridine synthase
	YFL002C	Putative ATP-dependent RNA helicase
	YFL003C	Protein involved in meiotic recombination
	YFL012W	Putative protein of unknown function
	YFL013C	Subunit of the INO80 chromatin remodeling complex
	YFL017W-A	Core Sm protein Sm G
	YFL021W	Transcriptional activator of nitrogen catabolite repression genes
	YFL023W	Unconventional prefoldin protein involved in translation initiation
	YFL024C	Subunit of NuA4, an essential histone H4/H2A acetyltransferase complex
	YFL025C	GPI inositol deacylase of the endoplasmic reticulum (ER)
	YFL027C	GTPase-activating protein for yeast Rab family members
	YFR002W	Linker nucleoporin component of the nuclear pore complex (NPC)
	YFR005C	Conserved zinc-finger domain protein involved in pre-mRNA splicing
	YFR006W	Putative X-Pro aminopeptidase
	YFR015C	Glycogen synthase
	YFR019W	1-phosphatidylinositol-3-phosphate 5-kinase
	YFR022W	Alpha-arrestin involved in ubiquitin-dependent endocytosis
	YFR040W	Protein required for function of the Sit4p protein phosphatase
	YGL006W	Vacuolar Ca2+ ATPase involved in depleting cytosol of Ca2+ ions
	YGL008C	Plasma membrane P2-type H+-ATPase
	YGL009C	Isopropylmalate isomerase
	YGL013C	Transcription factor that regulates the pleiotropic drug response
	YGL014W	Member of the PUF protein family
	YGL015C	Formin inhibitor
	YGL016W	Karyopherin beta
	YGL023C	Phosphatidylinositol 3-phosphate binding protein
	YGL027C	Processing alpha glucosidase I
	YGL028C	Cell wall protein with similarity to glucanases
	YGL035C	Transcription factor involved in glucose repression
	YGL039W	Aldehyde reductase
	YGR014W	Mucin family member involved in various signaling pathways
	YHR211W	Lectin-like cell wall protein (flocculin) involved in flocculation
	YIL001W	Putative protein of unknown function
	YIL005W	ER protein with chaperone and co-chaperone activity

	YIL007C	Evolutionarily conserved 19S regulatory particle assembly-chaperone
	YIL017C	GID Complex subunit, serves as adaptor for regulatory subunit Vid24p
	YIL019W	Protein required for pre-rRNA processing
	YIL023C	Zinc transporter
	YIL036W	Basic leucine zipper (bZIP) transcription factor from ATF/CREB family involved in stress-responsive regulatory network
	YIL037C	Pheromone-regulated protein
	YIL038C	Component of the CCR4-NOT core complex, involved in mRNA decapping
	YIL041W	BAR domain protein that localizes to early and late Golgi vesicles
	YIL042C	Mitochondrial protein kinase
	YIR001C	Cytoplasmic RNA-binding protein
	YIR003W	Subunit of a complex that associates with actin filaments
	YIR006C	Part of actin cytoskeleton-regulatory complex Pan1p-Sla1p-End3p
	YIR007W	Steryl-beta-glucosidase with broad specificity for aglycones
	YIR008C	Subunit of DNA primase
	YIR010W	Essential component of the outer kinetochore MIND complex
	YIR018W	Basic leucine zipper (bZIP) iron-sensing transcription factor
	YJL001W	Beta 1 subunit of the 20S proteasome
	YJL002C	Alpha subunit of the oligosaccharyltransferase complex of the ER lumen
	YJL010C	Essential subunit of U3-containing 90S preribosome
	YJL025W	Component of the core factor (CF) rDNA transcription factor complex
	YJL027C	Putative protein of unknown function
	YJL029C	Component of the GARP (Golgi-associated retrograde protein) complex
	YJL042W	Microtubule-associated protein involved in microtubule organization
	YJL052W	Glyceraldehyde-3-phosphate dehydrogenase (GAPDH), isozyme 1
	YJL054W	Component of the mitochondrial TIM22 complex
	YJL056C	Zinc-regulated transcription factor
	YJL057C	Protein kinase of unknown cellular role
	YJL058C	Subunit of TORC2 membrane-associated complex
	YJL060W	Kynurenine aminotransferase
	YJL063C	Mitochondrial ribosomal protein of the large subunit

	YJL073W	DnaJ-like chaperone required for nuclear membrane fusion during mating
	YJL074C	Subunit of the multiprotein cohesin complex
	YJL078C	GPI-anchored cell wall protein involved in export of sterols
	YJL080C	Essential RNA-binding G protein effector of mating response pathway
	YJL082W	Protein required for clearance of inclusion bodies
	YJL083W	EH domain-containing protein
	YJL089W	C6 zinc cluster transcriptional activator
	YJL094C	Putative K+/H+ antiporter
	YJL095W	MAPKKK acting in the protein kinase C signaling pathway
	YJL098W	Protein that forms a complex with the Sit4p protein phosphatase
	YJL099W	Member of the ChAPs (Chs5p-Arf1p-binding proteins) family
	YJL100W	Type II phosphatidylinositol 4-kinase
	YJL102W	Mitochondrial elongation factor involved in translational elongation
	YJL128C	MAP kinase kinase of the HOG signaling pathway
	YJL129C	Component of the Trk1p-Trk2p potassium transport system
	YJL130C	Bifunctional carbamoylphosphate synthetase/aspartate transcarbamylase
	YJR002W	Component of the SSU processome and 90S preribosome
	YJR005W	Beta-adaptin
	YJR008W	Protein of unknown function
	YJR013W	Glycosylphosphatidylinositol-alpha 1,4 mannosyltransferase I
	YJR016C	Dihydroxyacid dehydratase
	YJR017C	Peptidylprolyl-cis/trans-isomerase (PPIase)
	YJR024C	5'-methylthioribulose-1-phosphate dehydratase
	YJR031C	Guanine nucleotide exchange factor for ADP ribosylation factors (ARFs)
	YJR032W	Peptidyl-prolyl cis-trans isomerase (cyclophilin)
	YJR033C	Subunit of RAVE complex (Rav1p, Rav2p, Skp1p)
	YJR035W	Protein involved in transcription-coupled nucleotide excision repair
	YJR039W	Mitochondrial protein of unknown function
	YJR041C	Protein required for normal metabolism of the rRNA primary transcript
	YJR043C	Third subunit of DNA polymerase delta
	YJR051W	Fumarate reductase, catalyzes the reduction of fumarate to succinate
	YJR054W	Potassium transporter that mediates K^+ influx
	YJR061W	Protein required for N-glycan mannosylphosphorylation

	YJR062C	Amidase
	YJR066W	PIK-related protein kinase and rapamycin target
	YJR076C	Component of the septin ring that is required for cytokinesis
	YJR077C	Mitochondrial phosphate carrier
	YJR078W	Tryptophan 2,3-dioxygenase or indoleamine 2,3-dioxygenase
	YJR083C	Protein of unknown function
	YJR089W	Subunit of chromosomal passenger complex (CPC)
	YJR094C	Master regulator of meiosis that is active only during meiotic events
	YJR094W-A	Ribosomal 60S subunit protein L43B
	YJR106W	Protein involved in calcium homeostasis and exit from quiescence
	YJR107W	Putative lipase
	YJR109C	Large subunit of carbamoyl phosphate synthetase
	YJR117W	Highly conserved zinc metalloprotease
	YJR121W	Beta subunit of the F1 sector of mitochondrial F1F0 ATP synthase
	YJR125C	Protein containing an N-terminal epsin-like domain
	YJR132W	Karyopherin
	YJR134C	Protein of unknown function
	YJR136C	Subunit of the ASTRA complex, involved in chromatin remodeling
	YJR137C	Sulfite reductase beta subunit
	YJR138W	GTPase-activating protein (GAP) subunit of the Iml1p/SEACIT complex
	YJR139C	Homoserine dehydrogenase (L-homoserine:NADP oxidoreductase)
	YJR149W	Putative protein of unknown function
	YJR151C	Cell wall mannoprotein
	YJR152W	Allantoate permease
	YJR153W	Endo-polygalacturonase
	YJR154W	Putative protein of unknown function
	YJR155W	Putative aryl-alcohol dehydrogenase
	YKR002W	Poly(A) polymerase
	YKR008W	Component of the RSC chromatin remodeling complex
	YKR009C	3-hydroxyacyl-CoA dehydrogenase and enoyl-CoA hydratase
	YKR011C	Protein of unknown function
	YKR018C	Protein of unknown function
	YKR019C	EH domain-containing protein
	YKR021W	Alpha arrestin, substrate of calcineurin
	YKR022C	Essential protein that forms a dimer with Ntr1p
	YKR029C	Defining member of the SET3 histone deacetylase complex

	YKR054C	Cytoplasmic heavy chain dynein
	YLL019C	Protein kinase involved in negative regulation of PolIII transcription
	YLL021W	Component of the polarisome
	YLR002C	Subunit of a nuclear complex with Noc2p and pre-replicative complexes
	YLR006C	Cytoplasmic phosphorelay intermediate osmosensor and regulator
	YLR014C	Zinc finger transcription factor
	YLR015W	Subunit of COMPASS (Set1C) complex
	YLR020C	Steryl ester hydrolase
	YLR021W	Component of a heterodimeric Poc4p-Irc25p chaperone
	YLR024C	Cytoplasmic ubiquitin-protein ligase (E3)
	YLR027C	Cytosolic aspartate aminotransferase involved in nitrogen metabolism
	YLR032W	DNA helicase/Ubiquitin ligase
	YLR039C	Protein involved in retrograde transport to the cis-Golgi network
	YLR059C	3'-5' RNA exonuclease
	YLR068W	Nucleolar protein required for maturation of 18S rRNA
	YLR069C	Mitochondrial elongation factor involved in translational elongation
	YLR082C	Protein of unknown function
	YLR084C	Protein required for the maintenance of bud site selection
	YLR087C	Protein with structural similarity to lipid transport protein Vps13p
	YLR106C	Huge dynein-related AAA-type ATPase (midasin)
	YLR108C	Protein of unknown function
	YLR113W	Mitogen-activated protein kinase involved in osmoregulation
	YLR114C	Conserved protein involved in exocytic transport from the Golgi
	YLR115W	Subunit of the mRNA cleavage and polyadenlylation factor (CPF)
	YLR116W	Component of commitment complex
	YLR119W	Component of the ESCRT-I complex
	YLR120C	Aspartic protease
	YLR121C	Aspartic protease
	YLR126C	Putative glutamine amidotransferase
	YLR129W	Nucleolar protein
	YLR131C	Transcription factor required for septum destruction after cytokinesis
	YLR136C	mRNA-binding protein expressed during iron starvation
	YLR138W	Na+/H+ antiporter
	YLR144C	Intracellular beta-1,3-endoglucanase

	YLR145W	Subunit of RNase MRP
	YLR146C	Spermine synthase
	YLR148W	Component of CORVET membrane tethering complex
	YML017W	Asn rich cytoplasmic protein that contains RGG motifs
	YML018C	Protein of unknown function
	YML043C	Component of the core factor (CF) rDNA transcription factor complex
	YML046W	U1 snRNP protein involved in splicing
	YML059C	Serine esterase
	YML061C	DNA helicase, potent G-quadruplex DNA binder/unwinder
	YML064C	GTPase involved in initiation of Mitotic Exit Network (MEN)
	YML065W	Largest subunit of the origin recognition complex
	YML068W	Protein that modulates the efficiency of translation termination
	YML071C	Component of the conserved oligomeric Golgi complex
	YML074C	Nucleolar peptidyl-prolyl cis-trans isomerase (PPIase)
	YML076C	Homodimeric Zn2Cys6 zinc finger transcription factor
	YML091C	Protein subunit of mitochondrial RNase P
	YML093W	Subunit of U3-containing Small Subunit (SSU) processome complex
	YML097C	Guanine nucleotide exchange factor (GEF) and ubiquitin receptor
	YML099C	Zinc finger transcription factor involved in arginine-responsive genes
	YML102W	Subunit of chromatin assembly factor I (CAF-1), with Rlf2p and Msi1p
	YML103C	Subunit of the inner ring of the nuclear pore complex (NPC)
	YNL108C	Protein phosphatase
	YNL271C	Formin
	YNR006W	Endosomal protein that forms a complex with Hse1p
	YNR013C	Low-affinity vacuolar phosphate transporter
	YMR020W	Polyamine oxidase
	YMR086W	Component of eisosome required for proper eisosome assembly
	YMR152W	Aldehyde reductase
	YOL001W	Cyclin
	YOL004W	Component of both the Rpd3S and Rpd3L histone deacetylase complexes
	YOR005C	DNA ligase required for nonhomologous end-joining (NHEJ)
	YOR011W	Plasma membrane sterol transporter of the ATP-binding cassette family
	YOR014W	B-type regulatory subunit of protein phosphatase 2A (PP2A)

	YOR019W	Protein of unknown function
	YOR032C	bHLH protein with similarity to myc-family transcription factors
	YOR038C	Subunit of HIR nucleosome assembly complex
	YPL001W	Catalytic subunit of the Hat1p-Hat2p histone acetyltransferase complex
	YPL004C	Eisosome core component
	YPL009C	Component of the Ribosome Quality Control (RQC) complex
	YPL043W	Nucleolar protein
	YPL054W	Zinc-finger protein of unknown function
	YPL055C	Protein involved in histone H2B ubiquitination
	YPL057C	Mannosylinositol phosphorylceramide (MIPC) synthase catalytic subunit
	YPL078C	Subunit b of the stator stalk of mitochondrial F1F0 ATP synthase
	YPR003C	Putative sulfate permease
	YPR005C	Cytoplasmic protein involved in halotolerance
	YPR006C	2-methylisocitrate lyase of the mitochondrial matrix
	YPR011C	Mitochondrial transporter
